# Bayesian validation framework for dynamic epidemic models

**DOI:** 10.1016/j.epidem.2021.100514

**Published:** 2021-10-30

**Authors:** Sayan Dasgupta, Mia R. Moore, Dobromir T. Dimitrov, James P. Hughes

**Affiliations:** Fred Hutchinson Cancer Research Center, Seattle WA 98122, USA

**Keywords:** Epidemiological model validation, Markov Chain Monte Carlo, Bayesian credible interval, HIV transmission model

## Abstract

Complex models of infectious diseases are used to understand the transmission dynamics of the disease, project the course of an epidemic, predict the effect of interventions and/or provide information for power calculations of community level intervention studies. However, there have been relatively few opportunities to rigorously evaluate the predictions of such models till now. Indeed, while there is a large literature on calibration (fitting model parameters) and validation (comparing model outputs to data) of complex models based on empirical data, the lack of uniformity in accepted criteria for such procedures for models of infectious diseases has led to simple procedures being prevalent for such steps. However, recently, several community level randomized trials of combination HIV intervention have been planned and/or initiated, and in each case, significant epidemic modeling efforts were conducted during trial planning which were integral to the design of these trials. The existence of these models and the (anticipated) availability of results from the related trials, provide a unique opportunity to evaluate the models and their usefulness in trial design. In this project, we outline a framework for evaluating the predictions of complex epidemiological models and describe experiments that can be used to test their predictions.

## 1. Introduction

Complex nonlinear simulation models have been widely used in applied scientific disciplines, including the study of climate, geophysics, soil and air pollution, epidemiology, ecology, and other fields (see for example Caswell, 1976; Guttorp and Walden, 1987; Sampson and Guttorp, 1999; Poole and Raftery, 2000). Different types of models are available for this purpose, including stochastic, agent-based and deterministic differential equation models, the choice of which must be suited to the question of interest (see Halloran et al., 2017). While stochastic and agent based models incorporate random variation adding individual interactions such as partnership formation/dissolution, differential equation models are typically simpler and run faster, and are quite useful for analyses that require multiple runs of the model.

These models have also dominated the field of epidemic modeling, especially in complex diseases like HIV/AIDS (Johnson and White, 2011). In HIV epidemiology, these complex, dynamic models of infectious disease are used to understand the transmission dynamics of the disease, to obtain estimates and short-term projections of HIV epidemics (see Stanecki et al., 2012; Dorrington et al., 2005), to predict the effect of interventions (Case et al., 2018; de Montigny et al., 2018), to evaluate program implementation costs (Eaton et al., 2014; Schwartlander et al., 2011), to interpret clinical trial results (Adamson et al., 2019; Dimitrov et al., 2019) and to provide information for power calculations of community level intervention studies (Hayes and Moulton, 2017). For example, the increase in availability of antiretroviral therapy (ART) to individuals with HIV (WHO, 2013; Johnson et al., 2013) has opened up questions about the trajectory of the epidemic in presence of ART, including the probability of, and the level of coverage necessary for, complete eradication of the disease. To answer these questions, several models have been proposed to quantify the short and long term consequences of widespread ART availability and use on HIV prevalence, incidence and mortality, and to assess the level of coverage of ART necessary to substantially reduce or eliminate HIV (Granich et al., 2009; Kretzschmar et al., 2013).

Validity of results from a model is greatly dependent on the accuracy of the model structure and the input parameters, so a key goal during model development is to quantify and reduce the uncertainty about the structure and select model parameters which reproduce available epidemic data, using all of the available sources of evidence. This step is called calibration, and it involves careful tuning of the model structure to epidemiological endpoints, informed by available knowledge in the literature, and establishes credibility of the model (Ramin and Arhonditsis, 2013). Another important step in epidemiological model building is model validation, which is defined as a set of methods that help assess a model’s performance in making predictions. There are different types of validation processes, including face validity (the extent to which a model and its assumptions correspond to the current science as judged by experts), internal validity (whether the model behaves as intended and has been properly implemented), cross validity (how the model behaves when calibrated to one part of the data and used to predict the other), and external validity (to compare the predicted outcomes to measurable results). Each type of validation has its own sets of methods, strengths, limitations, and best practices (see Eddy et al., 2012).

For a clinical trial that was conducted based on insights gained from a model(s) in the planning phase, it can be quite useful to check how accurately the model(s) predicted the outcomes of interest when the trial is over. This is a possible external validation step. It is also quite useful to see if any source of bias in the model can be re-accounted for, and whether the model structure can be recalibrated to ensure its re-usability in the future (Eddy et al., 2012). The results of these analyses can help investigators determine applicability of the model in the public-health decision making process, and in planning of future trials.

In this article, we propose a framework for model comparison and evaluation based on observed data. To be precise, we are interested in external validation (will be called validation hereon) and re-calibration of a model, based on observed data. It is clear that the most powerful way to validate the prediction of a mathematical model is to record the model and its predictions in advance of new observations. Recently several community level randomized trials of combination HIV intervention have been planned and/or initiated (Boily et al., 2012). Significant epidemic modeling efforts were conducted during trial planning and became integral to the design of these trials. The existence of these models that have been designed to predict trial results in a specific setting, and the (anticipated) availability of results from those trials, provides a unique opportunity to evaluate those models and their usefulness in trial design through an external validation step. In this type of analysis, the goal is to test and potentially validate the model predictions of intervention with the trial results at the end of the trial. If the trial results are similar to the model predictions, then this validates the model for the trial in question, and the model can be used for further analyses with more confidence (Boily et al., 2012).

Interestingly, there have been relatively few such opportunities to rigorously evaluate the predictions of HIV epidemic models until now. Indeed, while there are multiple theoretical methods for calibration and external validation of complex models (see Bayarri et al., 2007; Kennedy and O’Hagan, 2001; Poole and Raftery, 2000), fairly simple procedures have been used for calibration and validation of models partially due to lack of substantial high quality, population-level data on HIV incidence, HIV prevalence and sexual behavior. The main goal of this article is to develop a step-by-step methodological framework for model validation, comparison and selection, when detailed data are available. Our approach takes into account the key uncertainties in model parameters, allowing for subsequent recalibration of the model, if needed and/or possible. An additional objective is to determine the extent to which external data can be used to reject a complex epidemiological model. The key components of the approach include the use of Gaussian process response-surface method, and introduction of Bayesian representations of model bias and uncertainty, following the works of Bayarri et al. (2007), Kennedy and O’Hagan (2001) and Kennedy et al. (2002).

In Section 2, we formulate the problem and propose our methodological framework for validation of a dynamic epidemic model based on the Bayesian validation framework of Bayarri et al. (2007). Additionally, we propose a way to make decisions regarding model fit based on Bayesian credible intervals. In Section 3, we describe a mathematical model (*M*_*W*_) of a heterosexual HIV epidemic originally considered in Woods et al. (2018) to investigate how the proportion of early transmission affects the impact of ART on reducing HIV incidence. The model includes stages of HIV infection, flexible sexual mixing, and changes in risk behavior over the epidemic, and was calibrated to HIV prevalence data from South Africa. Next, we apply the proposed methodology to evaluate the performance of this model under departures of different model assumptions. In other words, we try to falsify this model under null and alternative conditions, and note how it performs in such situations. In Section 4, we discuss the implications of our results. Some additional results are provided in Appendix.

## 2. Methods

### 2.1. Model framework

#### 2.1.1. Epidemiological model definition

In this article, we consider epidemiological models of the form *y*_*M*_ *= M*(*x, θ*). Here *y*_*M*_ ∈ *Ω*_*M*_, the model output, typically emulates a population level outcome of interest, such as HIV prevalence. Each entry in *y*_*M*_ represent the projected outcome at different times within different demographics or different risk subgroups. The model *M* is a complex non-linear function, typically defined as a solution to a system of ordinary differential equations, which translates a vector of community-specific parameters *x* ∈ *Ω*_*x*_ and a vector of global parameters *θ* ∈ *Ω*_*θ*_ into the outcome *y*_*M*_.

Throughout this paper a community will be any population within which an epidemic can be modeled independently from other communities. A community may be all individuals in a given city or region or may only be a subset, such as people who inject drugs, within which the epidemic may be evolving independently. A community may be divided into several interconnected sub-populations, for example divided by demography.

The community-specific parameters *x* (a vector of size *p*_*x*_) denote information about attributes of a given community within which we wish to calculate the model output *y*_*M*_. In the context of HIV prevention trials, *y* can be the projected HIV prevalence by gender and age group, and *x* might include community-specific information about level of ART usage, initial testing coverage (that differ by subgroups or communities), HIV prevalence for that site at some previous time point. The joint spaces *Ω*_*x*_ and *Ω*_*M*_ together represent the granularity of the model, the class of all possible sub-populations and communities for which the model *M*, after calibration, can predict the population-level outcome separately.

The ‘global’ parameters *θ* (of size *p*_*θ*_) describe the part of the model that is universal, or fixed across different communities. For example, in the context of a HIV trial, *θ* contain various biologic, behavioral or intervention characteristics (e.g., parameters for intervention efficacy, transmission risk per act, sexual mixing, background level of male circumcision etc.) which are shared between community-specific simulations.

The components of *y*_*M*_, *x*, and *θ* depend on the modeling context and intended granularity. For example, it may be crucial for a model on HIV transmission to predict the response separately for men who have sex with men (MSMs) and transgender women (TGW), two crucial sub-populations at risk for the disease. However that distinction may be inconsequential in the context of some other disease (say cancer), and a universal response may be preferred under a model aiming to predict the disease for these sub-populations. We define the ‘model structure’ of *M* to be the three spaces *Ω*_*x*_, *Ω*_*θ*_, and *Ω*_*M*_.

#### 2.1.2. Sources of error in the model

In principle, if *x* for a given sub-population and *θ* are known precisely, and the model is exactly ‘true’, then *y*_*M*_ or *M*(*x, θ*) should be able to predict the underlying truth without error. In practice, however, it is quite likely that there will be uncertainty regarding the true values of *x* and *θ*, and the model structure *M* is only a useful approximation of the underlying mechanics. Ideally, we would assess a model *M* by comparing the modeled epidemiological value *y*_*M*_ with the true value (denoted by *y*_*R*_), though this may not be possible, as the reality will have to be estimated with error from a sample of the population. All these sources of uncertainties must be considered in assessing the model predictions.

#### 2.1.3. Field data vs. reality

Suppose we are interested in evaluating our model for *K* different subgroups, stratified under one or more baseline/longitudinal characteristics or communities, based on model projections at a given time during the study and the true outcome at that time. As mentioned before, we assume that the outcome is not observed directly, but it can be measured without bias. Let *y*_*R,k*_ be the true outcome for community *k*, *k* = 1,…*,K*, at the specified time, and assume that we observe *y*_*F,k*_, the ‘field data’ for this community at that time, as reality measured with error, that is,

(1) $$y_{F,k}=y_{R,k}+\epsilon^{F}\left(y_{R,k}\right),$$

where *ϵ*^*F*^ (*y*_*R,k*_) are mean 0 errors with variance 1∕*λ*_*F*_ (*y*_*R,k*_), for a precision process *λ*_*F*_ (·), which may or may not be explicitly known (we discuss this in details in Section 2.3.1). Further distributional assumptions can be made on *ϵ*^*F*^ (*y*_*R,k*_) under specific problem setups, for example, one operational assumption often used in validation problems is that *ϵ*^*F*^ (*y*_*R,k*_)’s are independent normal random errors (see Bayarri et al., 2007). However, one might have to consider transformations of the responses *y*_*R,k*_ and *y*_*F,k*_ to enable easier distributional assumptions and interpretation of the error function *ϵ*^*F*^ (·) in Eq. (1).

**Note:** Note that although *y*_*F*_ and *y*_*M*_ can be evaluated at several times during the study, we consider only one fixed time point here to compare the model projections with observed data, and as a result to simplify notations, we have dropped the suffix *t* from *y*_*F*_, *y*_*M*_ as well as *y*_*R*_.

#### 2.1.4. Induced prior for y_M_

Information about different parameters of a model are often available from previously conducted trials or relevant observational studies in the literature. Knowledge from these various sources are combined for the quantification of the community-specific and global parameters, and the model is then carefully calibrated, resulting in well-informed prior distributions for these quantities. Thus, in this article, we assume that *X*_*k*_,^1^ the community-specific parameters for the *k*th subgroup, and *Θ*, the global parameter vector, are random variables with distributions $G_{X_{k}}\left(\cdot ;\eta_{X_{k}}\right)$ and $H_{\Theta }\left(\cdot ;\eta_{\Theta }\right)$ respectively, and with underlying hyperparameters $\eta_{X_{k}}$ and *η*_*Θ*_. These distributions quantify the level of uncertainties about the different model inputs, the community-specific and global parameters. Also note, that the distributions of *X*_*k*_ and *Θ* jointly induce a distribution, denoted by $F_{X_{k},\Theta }^{M}$, on the random model function *M*(*X*_*k*_*,Θ*).

**Note:** Simplification of the above setup is possible too, for example, if information about *X*_*k*_ is available through high quality estimates ${\hat{x}}_{k}$, we can assume that the true underlying community-specific parameter set is known *apriori*, and $X_{k}={\hat{x}}_{k}$. Similarly, information about $H_{\Theta }$ can be available only as scalars, $\Theta =\hat{\theta }$, that are available as estimates/calibrated values for the ‘true’ structural parameters of the model. In such cases, we may choose to use these scalars directly as ‘true’ (or best known) values in the analysis.

### 2.2. Model discrepancy

#### 2.2.1. The discrepancy function, D_M_

Statistical assessment of a model prediction is based on the difference between the true value *y*_*R*_ and the model prediction. To that effect, we define the discrepancy function *D*_*M*_, which we will use throughout this article. For a given model *M* with an instance of community-specific parameters *x* and global parameters *θ*, the discrepancy between the model output and the relevant response process *y* is given as,

(2) $$D_{M}(y,x,\theta )=y-M(x,\theta ).$$

In our problem setup, as previously mentioned in Section 2.1.4, *X*_*k*_, the community-specific parameters for the *k*th subgroup, and *Θ*, the global parameter vector, are random variables, while the true response for is *y*_*R,k*_. Hence, the ‘random’ discrepancy function for subgroup *k* becomes *D*_*M*_(*y*_*R,k*_*,X*_*k*_*,Θ*) = *y*_*R,k*_ − *M*(*X*_*k*_*,Θ*). We can analyze the distribution of *D*_*M*_(*y*_*R,k*_) for *k* = 1,…*,K* (for each of the different subgroups/communities) to evaluate model performance. The variation in *D*_*M*_(*y*_*R,k*_) between these communities can be useful in identifying areas where the model has failed to adequately capture the effect of factors on the response.

**Note:** Although we are interested in learning about *D*_*M*_(*y*_*R,k*_), in the absence of concrete knowledge about *y*_*R*_, we typically have to rely on *D*_*M*_(*y*_*F,k*_) to infer about this quantity.

#### 2.2.2. A Bayesian approach to evaluate D_M_

Since *D*_*M*_(*X*_*k*_*,Θ*) is a function of the random variables *X*_*k*_ and *Θ*, for which we have distributions of the form of $G_{X_{k}}\left(\cdot ;\eta_{X_{k}}\right)$ and $H_{\Theta }\left(\cdot ;\eta_{\Theta }\right)$, one trivial way to obtain and analyze the distribution of *D*_*M*_ is through the transformation *D*_*M*_(*X*_*k*_*,Θ*) = *y*_*R,k*_ − *M*(*X*_*k*_*,Θ*) on the induced distribution $F_{X_{k},\Theta }^{M}$. Although the true response *y*_*R,k*_ is typically unknown, one can replace *y*_*R,k*_ by its observed estimate *y*_*F,k*_. Although the resultant distribution is well-defined, it can only be used to assess the model in its current state. However, our interests may also lie in identifying the possible sources of error in the model, and if possible, recalibrate these faulty parts, based on observed data, to improve model performance.

Hence, using a Bayesian analysis described in the next section (Section 2.3), we propose to estimate the posterior distribution of the community-specific parameters *X*_*k*_, global parameters *Θ*, and the discrepancy function *D*_*M*_(*X*_*k*_*,Θ*), to assess the model for community *k* and identify its sources of discrepancy. Ideally, the distribution of *D*_*M*_ should be centered around zero with small variance. We will show how to quantitatively evaluate this property, even in high dimensions using the idea of posterior tail probability (or *p*_*T P*_). We will also show how to use this procedure to identify the different sources of error or bias in model parameters, both community-specific and global, and model structure.

#### 2.2.3. Gaussian Stochastic Processes (GaSP) model as a prior for D_M_

We define a functional prior $P_{D_{M}}(x,\theta )$ on *D*_*M*_(*x, θ*) for each pair (*X* = *x,Θ* = *θ*) using a Gaussian Stochastic Process (GaSP). A GaSP is useful in modeling functional outputs like *D*_*M*_ that depend smoothly on its arguments, *x* and *θ* (see Sacks et al., 1989; Currin et al., 1991). Although a GaSP is most helpful when all of the model arguments are continuous, it can also handle cases when one or more (but not all) of its arguments are discrete, by specifying a separate Gaussian response surface at each level of the discrete factors. This, combined with the priors on *X* and *Θ*, produces a prior for *D*_*M*_(*X,Θ*).

Consider our setup of *K* subgroups (or communities), with community-specific random variables *X*_*k*_ and a global random variable *Θ*. Suppose we want to run the model *M* for $X_{k}={\tilde{x}}_{k}$, for *k* ∈ {1,…*,K*}, and $\Theta =\tilde{\theta }$, and evaluate the discrepancy of the model for these subgroups for the given realizations. Define the *K* × 1 vector of discrepancies for the *K* subgroups as $d_{M}={\left(D_{M}\left({\tilde{x}}_{1},\tilde{\theta }\right),\ldots ,D_{M}\left({\tilde{x}}_{K},\tilde{\theta }\right)\right)}^{T}$. Let *Z*_*k*_ denote the multivariate random variable *Z*_*k*_ = (*X*_*k*_*,Θ*), such that $z:={\left(z_{1}^{T},\ldots ,z_{K}^{T}\right)}^{T}$ forms a *K* × *p*_*z*_ matrix, where *p*_*z*_ = *p*_*x*_ + *p*_*θ*_ and $z_{k}=\left({\tilde{x}}_{k},\tilde{\theta }\right)$ is a realization of *Z*_*K*_. The GaSP formulation assigns a *K*-variate Gaussian distribution for the vector **d**_*M*_ with mean function $\mu_{d}(z)={\left(\mu_{d}\left(z_{1}\right),\ldots ,\mu_{d}\left(z_{K}\right)\right)}^{T}$. The covariance function *C*_*d*_(·,·) for the Gaussian process is given as,

(3) $$C_{d}\left(z_{k},z_{l}\right)=\frac{1}{\lambda_{d}}\exp\left(-\sum_{j=1}^{p_{z}}\beta_{d,j}{\left|z_{k,j}-z_{l,j}\right|}^{\alpha_{d,j}}\right)$$

for parameters $\beta_{d}=\left\{\beta_{d,1},\ldots ,\beta_{d,p_{z}}\right\}$ and $\alpha_{d}=\left\{\alpha_{d,1},\ldots ,\alpha_{d,p_{z}}\right\}$ that control the amount of correlation between *D*_*M*_(*z*_*k*_) and *D*_*M*_(*z*_*l*_), and with *λ*_*d*_ controlling the precision of the Gaussian surface. Note that for elements $z_{k}=\left({\tilde{x}}_{k},\tilde{\theta }\right)$ and $z_{l}=\left({\tilde{x}}_{l},\tilde{\theta }\right)$ (where *k,l* ∈ {1,…*,K*}), the last *p*_*θ*_ elements in the sum $\sum_{j=1}^{p_{z}}\beta_{d,j}{\left|z_{k,j}-z_{l,j}\right|}^{\alpha_{d,j}}$ are 0, as $z_{k,j+p_{x}}=z_{l,j+p_{x}}={\tilde{\theta }}_{j}$ for *j* = 1,…*,p*_*θ*_. Hence the above simplifies to $C_{d}\left(z_{k},z_{l}\right)=\frac{1}{\lambda_{d}}\exp\left(-\sum_{j=1}^{p_{x}}\beta_{d,j}{\left|x_{k,j}-x_{l,j}\right|}^{\alpha_{d,j}}\right)$ and as a result only the first *p*_*x*_ elements of the hyperparameters *β*_*d*_ and *α*_*d*_ need to be specified.

Now let us briefly discuss the specifications of the hyperparameters *μ*_*d*_, *λ*_*d*_, *α*_*d*_ and *β*_*d*_. First, to limit the number of hyperparameters in the model, the components of *α*_*d*_ are fixed. For example, *α*_*d*_ = 2 gives us the standard Gaussian process formulation (see Bayarri et al., 2007), and for the rest of the article, we will consider the elements of *α*_*d*_ to be fixed at that value. The mean function of the GaSP, *μ*_*d*_(·), can be chosen to be fixed at a constant *μ*, the most intuitive choice being *μ* = 0. To generalize the analysis further, we can specify symmetric mean 0 distributions to be used as priors if required. For elements of *β*_*d*_, we can either specify priors for them though another set of hyperparameters, or fix them at estimates, determined using data driven methods. However, the precision parameter *λ*_*d*_ is typically kept stochastic, and we can specify priors like inverse gamma for *λ*_*d*_ with the hyperparameters (shape and scale) determined in a data-driven manner. Please see a more detailed discussion on how to choose these hyperparameters for a practical setting as our simulation example in Section 3.2.

### 2.3. Assessing the model

#### 2.3.1. Specification for λ_F_ in the Bayesian analysis

Often *λ*_*F*_ (*y*_*R,k*_) can be determined by the sampling design of the field data *y*_*F,k*_ for a given subgroup or community *k*. Consider the case of a epidemiological model that predicts HIV prevalence, where reality *y*_*R,k*_ is a proportion in the interval (0,1), and is estimated through sampling. Now if the observed ‘field data’ *y*_*F*_ is collected based on a set of *N* samples, then it will be distributed as $Ny_{F,k}\sim \mathrm{Bin}\left(N,y_{R,k}\right))$. Here the precision parameter *λ*_*F*_ has an explicit expression,

(4) $$\lambda_{F}\left(y_{R,k}\right)=\frac{1}{Var\left(y_{F,k}\right)}=\frac{N}{y_{R,k}\left(1-y_{R,k}\right)}.$$

Note that for a large enough sample size, *y*_*F,k*_ is approximately distributed as Gaussian with variance 1∕*λ*_*F*_ (*y*_*R,k*_). A natural estimate for *λ*_*F*_ (*y*_*R,k*_) can be produced by replacing *y*_*R,k*_ in (4) by its estimate *y*_*F,k*_. However in case the error structure is unknown or the estimates are unreliable, standard priors like 1∕*λ*_*F*_ can be used if replicates are available, otherwise data dependent priors centered at a suitably defined estimate of *λ*_*F*_ (*y*_*R,k*_) can be used in the analysis instead.

#### 2.3.2. Calculation of posterior of D_M_

Using the multivariate (*K*-variate) prior for *D*_*M*_, the induced model prior $F_{X_{k},\Theta }^{M}$, and the error process *λ*_*F*_, we evaluate the posterior distribution of *D*_*M*_ at each of the *K* communities. Below we give details for the full Bayesian analysis. This analysis can be further modified based on different distributional assumptions on the various parts of the model (community-specific and/or global) and the observed data (error variance). One such modified analysis has been discussed in the Appendix, which was used in our simulation exercise. From here on, we will also assume that *ϵ*^*F*^ (*y*_*R,k*_)’s are independent normal random errors. This assumption is certainly valid in many cases of epidemiological modeling, when model predictions are of standard forms (for example, binomial proportions like prevalence or rates like log-incidence) and the field data estimates are obtained using survey data or other standard methods, as the estimation error is approximately Gaussian due to the Central Limit Theorem. This is the case in our simulation example that we will study in detail later.

Suppose we want to run the analysis for *K* different communities with random community-specific parameters for the *k*th community, *X*_*k*_, with $X_{k}\sim G_{X_{k}}$, and a random global parameter set *Θ*, with distribution $H_{\Theta }$. Recall the equations that connect *y*_*R,k*_*, y*_*F,k*_ and *M*; for community *k*,

$$y_{F,k}=y_{R,k}+\epsilon^{F}\left(y_{R,k}\right),$$

$$y_{R,k}=M\left(X_{k},\Theta \right)+D_{M}\left(X_{k},\Theta \right),$$

(5) $$\text{where}\;\epsilon^{F}\left(y_{R,k}\right)\sim N\left(0,1/\lambda_{F}\left(y_{R,k}\right)\right).$$

Also, suppose that the precision process is unknown, and we have prior distributions of the form *λ*_*F*_ (*y*_*R,k*_) ~ *P*(*λ*_*F*_ |*y*_*R,k*_) for community *k*. Given the unknowns, these produce a multivariate normal density for the collection of all field data *y*_*F,k*_ for *k* ∈ {1,…*,K*}, denoted by $f(y_{F,1},\ldots ,y_{F,K}|\Theta ,X_{1},\ldots ,X_{K},\lambda_{F}\left(y_{R,1}\right),\ldots ,\lambda_{F}\left(y_{R,K}\right)$,$D_{M}\left(X_{1},\Theta \right),\ldots ,D_{M}\left(X_{K},\Theta \right),\lambda_{d},\mu_{d},\beta_{d},\alpha_{d})$. Removing the known (or fixed) quantities from the likelihood, the posterior density of the unknowns given the data *y*_*F,k*_ for *k* ∈ {1,…*,K*} can be written as

(6) $$P(\Theta ,X_{1},\ldots ,X_{K},\lambda_{F}\left(y_{R,1}\right),\ldots ,\lambda_{F}\left(y_{R,K}\right),D_{M}\left(X_{1},\Theta \right),\ldots ,D_{M}\;\;\times \left(X_{K},\Theta \right),\lambda_{d}|y_{F,1},\ldots ,y_{F,K})\;\propto f(y_{F,1},\ldots ,y_{F,K}|\Theta ,X_{1},\ldots ,X_{K},\lambda_{F}\left(y_{R,1}\right),\ldots ,\lambda_{F}\left(y_{R,K}\right),\;\;D_{M}\left(X_{1},\Theta \right),\ldots ,D_{M}\left(X_{K},\Theta \right),\lambda_{d})\;\;P(\Theta ,X_{1},\ldots ,X_{K},\lambda_{F}\left(y_{R,1}\right),\ldots ,\lambda_{F}\left(y_{R,K}\right),\;\;D_{M}\left(X_{1},\Theta \right),\ldots ,D_{M}\left(X_{K},\Theta \right),\lambda_{d}).$$

Note that the prior $P(\Theta ,X_{1},\ldots ,X_{K},\lambda_{F}\left(y_{R,1}\right),\ldots ,\lambda_{F}\left(y_{R,K}\right)$, $D_{M}\left(X_{1},\Theta \right),\ldots ,D_{M}\left(X_{K},\Theta \right),\lambda_{d})$ can be written as

(7) $$P\left(\Theta ,X_{1},\ldots ,X_{K},\lambda_{F}\left(y_{R,1}\right),\ldots ,\lambda_{F}\left(y_{R,K}\right),D_{M}\left(X_{1},\Theta \right),\ldots ,D_{M}\left(X_{K},\Theta \right),\lambda_{d}\right)\;=H_{\Theta }G_{X_{1}}\ldots G_{X_{K}}P\left(\lambda_{F}|y_{R,1}\right)\ldots P\left(\lambda_{F}|y_{R,K}\right)P\left(\lambda_{d}\right)P\left(D_{M}|\Theta ,X_{1},\ldots ,X_{K},\lambda_{d}\right),$$

where $P\left(D_{M}|\Theta ,X_{1},\ldots ,X_{K},\lambda_{d}\right)$ is the multivariate GASP prior for the discrepancy at all *K* communities. From here onward, we will refer to the *k*-variate discrepancy random variable as ${\text{D}}_{M}\;:=\left\{D_{M}\left(X_{1},\Theta \right),\ldots ,D_{M}\left(X_{K},\Theta \right)\right\}$.

The posterior distribution can be determined through MCMC techniques (Robert and Casella, 2004). The MCMC step can be performed in a number of different ways. In our simulation study, we have used the Metropolis Hastings (MH) algorithm to calculate the posterior distribution, however alternative techniques like Bayesian Melding (see Poole and Raftery, 2000), Hamiltonian Monte Carlo or HMC (see Chatzilena et al., 2019; Betancourt, 2017) can be used instead. For example, in comparison with the traditional MH algorithm, HMC can offer greater computational efficiency, especially in higher dimensional or more complex modeling situations. Please also see Robert and Changye (2020) for a comparative analysis of these methods to determine the most optimal framework for a given problem. In the Appendix, we describe the MH procedure for a simplified setup, which will be the setup for the simulation example presented in Section 3.

#### 2.3.3. Identifying sources of error

**Fig. 1.**
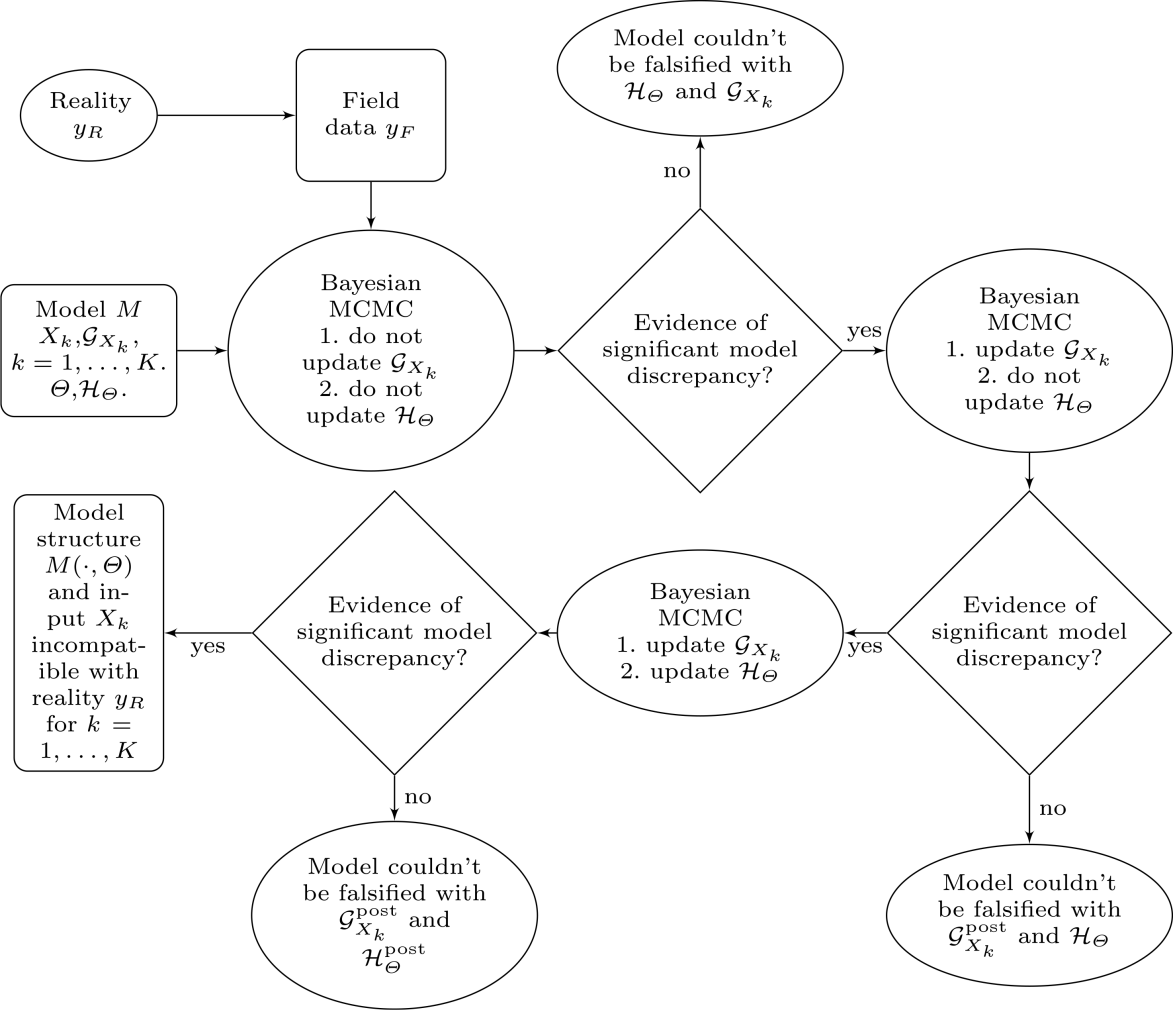
One possible schematic of the Bayesian analysis for model validation.

The Bayesian procedure employed to obtain the posterior distribution of discrepancy vector $D_{M}=\left\{D_{M}\left(X_{1},\Theta \right),\ldots ,D_{M}\left(X_{K},\Theta \right)\right\}$ can be run in a number of different ways depending on the targeted goals of the validation procedure, as described below. We first describe what we mean by ‘updating a prior’ in this context. Note that the main goal of our Bayesian analysis is to obtain the posterior distribution of **D**_*M*_. This posterior distribution is informed by the priors on **D**_*M*_, the model structure *M*, the stochastic distributions $G_{X_{k}}$ on *X*_*k*_ for *k* ∈ {1,…*,K*} and $H_{\Theta }$ on *Θ*, and the observed data *y*_*F,k*_ for *k* ∈ {1,…*,K*}. Now, in the process of obtaining the posterior distribution of **D**_*M*_, we can additionally choose to obtain the posterior distributions of any, some or all of the random variables *X*_1_,…*,X*_*K*_*,Θ*. For parameters that we choose to update, their stochastic distributions are considered as priors and the relevant posteriors are obtained through the MCMC procedure. Parameters which are not being updated are not considered directly in the MCMC procedure. While, we do have to generate realizations of these parameters from their current stochastic distributions for calculating the likelihood in each MCMC update. Their distributions are not updated in the MCMC steps. In the Appendix, we have described how to achieve this in our simulation setup through the Metropolis Hastings procedure. Although the flow chart in Fig. 1 shows the flow of the analysis when the objective is to re-calibrate all parameters (if necessary to obtain a better model fit), Researchers may be interested in running only part of the analysis flow depending on study objectives. Below we present a few such ways to run the analysis:

1. **Evaluation of current model** To assess the model in its current state, we can run the analysis without updating the priors $H_{\Theta }$ or $G_{X}$. As a result we only obtain the posterior distribution of **D**_*M*_ along with the posteriors of the hyperparameters controlling the discrepancy distribution in the model.
2. **Recalibration of community-specific parameters** If we are interested in assessing the model after allowing for recalibration/re-tuning of the community-specific parameters, we can update $G_{X_{k}}$ to obtain $G_{X_{k}}^{\text{post}\;}$, the posterior distribution of *X*_*k*_ along with the posterior distribution of **D**_*M*_. Note that one might choose to update all of the community-specific parameters at a time together, or one by one, or in groups of more than one.
3. **Recalibration of community-specific and global parameters** If we are interested in assessing the model after allowing for recalibration of both the global and the community-specific parameters, we can choose to update $H_{\Theta }$ to obtain $H_{\Theta }^{\text{post}}$, the posterior distribution of *Θ* as well, along with $G_{X_{k}}^{\text{post}\;}$, the posterior distribution of *X*_*k*_ and the posterior distribution of **D**_*M*_. Like in step 2, one might choose to update any combination of community-specific and global parameters at a time together, or in steps (for which new data become available after the last model evaluation), or till they are all updated.

**Note:** The entire analysis can be conducted in a single step or in a series of several steps, with each step devoted to updating and analyzing a specific parameter or a group of parameters. The order in which the community-specific and global parameters are updated in the analysis above is interchangeable, depending on the context and necessity. For example, the order can be determined by running sensitivity analyses to gauge the influence of each parameter (or parameter set) on the final model outputs, and then updating them in order of importance.

After running the analysis in one of the aforementioned ways, the posterior distribution of the discrepancy, ***P***^post^(**D**_*M*_), is assessed carefully to see if it is considerably removed from being centered at 0, which may suggest (depending on the analysis run) that any or all of (i) the structure of the model, (ii) the priors for *Θ* and (iii) the priors for *X*_*K*_ need to be updated. A step by step flowchart for the entire scope of the analysis under the discussed setup is given in Fig. 1.

#### 2.3.4. Quantification of model performance with Posterior Tail Probability (p_T P_)

When the model assumptions are satisfied, that is, when we have informative and precise priors on *X*_1_,…*,X*_*K*_*,Θ* and the model is a close approximation of the reality, we expect the posterior distribution of the discrepancy to be roughly centered around **0**. However, under violation(s) of any one or more of these assumptions, the posterior distribution will either suffer a shift in mean, or show inflated variance, be multimodal, or any combination of the above. In this section, we will concern ourselves only with the first type of alternative, that is, when the posterior distribution suffers a mean shift. The goal is to quantify the extent of this shift as a model validation metric. Note that our interests may lie in validating the model for (i) all *K* communities together, (ii) for a given subset of these *K* communities, or (iii) for each community separately. We present methods for the second situation here, and it is easy to see that the first and the third situations can be viewed as special cases of the second.

Let us assume that we want to assess the discrepancy of the model *M* at *J* out of *K* communities, $\left\{X_{i_{1}},X_{i_{2}},\ldots ,X_{i_{J}}\right\}$ with *i*_1_*,i*_2_,…*,i*_*J*_ ∈ {1,2,…*,K*} and 1 ≤ *J* ≤ *K*. Let **D**_*M*_ be the multivariate random variable (of size *J* × 1) representing discrepancy at these communities. The Mahalonobis distance for a discrepancy vector **D**_*M*_ = **d** from a given center *μ* and a given covariance matrix *V* is

(8) $$\Delta_{\mu ,V}(d)=\sqrt{{\left(d-\mu \right)}^{\prime }V^{-1}(d-\mu )}$$

The Posterior Tail Probability (*p*_*T P*_) measures the probability that an observation drawn at random from the posterior distribution of **D**_*M*_ lies further away from the mean of that distribution than does **0**, the expected discrepancy under the null hypothesis that the Model is true. Effectively, it is a (two-sided) tail probability, calculated based on the relative location of **0** with respect to the posterior distribution of **D**_*M*_.

Let ***η*** and *V* be the posterior mean and covariance matrix for the posterior distribution for **D**_*M*_, and $\bar{d}$ is the sample mean of discrepancies from the MCMC analysis, while $\hat{V}$ is a sample estimate for *V*. Now note that the Mahalonobis distance for a discrepancy sample **D**_*M*_ = **d** from the center ***η*** and for the given covariance matrix *V* is given as

$$\Delta_{\eta ,V}(d)=\sqrt{{\left(d-\eta \right)}^{\prime }V^{-1}(d-\eta )}.$$

To estimate *p*_*T P*_ from the MCMC data, **d**_*j*_, we:

1. Calculate ***δ*** = {*δ*_1_,…*,δ*_*N*_}, where *δ*_*j*_ is the distance between sample **d**_*j*_ and $\bar{d}$. That is, $\delta_{j}=\sqrt{{\left(d_{j}-\bar{d}\right)}^{\prime }{\hat{V}}^{-1}\left(d_{j}-\bar{d}\right)}.$
2. Compute *δ*^0^, the distance between **0** and $\bar{d}$, as $\delta^{0}=\sqrt{{\bar{d}}^{\prime }{\hat{V}}^{-1}\bar{d}}$.
3. Calculate ${\hat{p}}_{TP}=\sum_{j=1}^{N}\left(\delta_{j}\geq \delta^{0}\right)/N$.

**Fig. 2.**
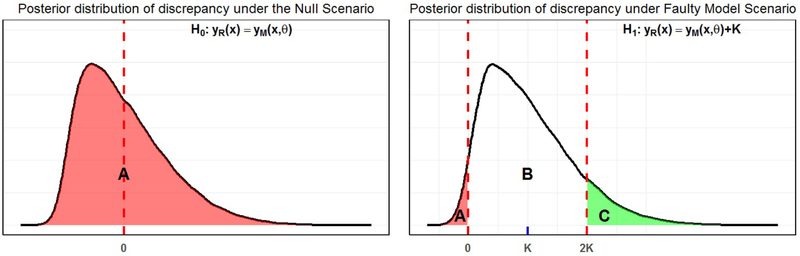
Posterior tail probabilities (*p*_*T P*_), as denoted by the colored regions, under the Null and an hypothetical Faulty model structure scenario for discrepancy at a single community: (i) Under the Null, the mean of the discrepancy distribution is 0 itself, so any random sample from the discrepancy distribution will have Mahalonobis distance from its center greater than that between 0 and the center, and thus the posterior tail probability region will cover the entire discrepancy distribution, as shown by the shaded region *A*. (ii) Under the alternative, when the center of the discrepancy is at *K* units away from 0, the Mahalonobis distance between a randomly chosen sample and the center will be greater than that between the center and 0 if the sample belongs to either of the shaded regions *A* and *C*, and will be lesser than that between the center and 0 if it belongs to region *B*. Thus the posterior tail probability region will cover regions *A* and *C.* (For interpretation of the references to color in this figure legend, the reader is referred to the web version of this article.)

Note that *p*_*T P*_ estimates the probability that $\Delta_{\eta ,V}\left(D_{M}\right)\geq \Delta_{\eta ,V}(0)$, that is,${\hat{p}}_{TP}=\mathbb{P}\left(\Delta_{\bar{d},\hat{V}}\left(D_{M}\right)\geq \Delta_{\bar{d},\hat{V}}(0)\right)$. Fig. 2 gives us an overview of how the posterior tail probability might look like when discrepancy is univariate (for a single community). Under *H*_0_, the true center (mean) of the posterior discrepancy is 0, hence the distance between the true center and 0 is also trivially 0, that is, *Δ*_***η***=0*,V*_ (0) = 0. Thus for any sample, its Mahalonobis distance from the true center (or its mean) will be greater than or equal to the distance between the true center and 0, and its posterior tail probability will be 1 (the probability under the shaded region *A* in the leftmost figure in Fig. 2). On the other hand, under a hypothetical alternative situation when the model is not valid (*H*_1_) and that the true center (or mean) is at *K*, the Mahalonobis distance between a randomly chosen sample and the center will be greater than that between the center and 0 if the sample belongs to either of the shaded regions *A* and *C*, and will be lesser than that between the center and 0 if it belongs to region *B*.

Now, observe from Fig. 2, that the area of the region *B* is exactly 1 − *p*_*T P*_, and that the interval that bounds it forms a credible interval of level *p*_*T P*_ for the posterior discrepancy around its posterior mean, that is, the interval (0,2*K*) is a $C_{p_{TP}}$ for the posterior discrepancy **D**_*M*_. Mahalonobis distance helps to extend this idea to multivariate discrepancy vectors, where *p*_*T P*_ gives us the minimum *α* level of a credible region centered around the multivariate posterior mean that does not contain **0**.

Because the MCMC chain consists of autocorrelated samples, we estimate $\hat{V}$ using an approach based on batch means Wakefield (see Chapter 3 of 2013), Glynn and Iglehart (see Chapter 3 of 1990). We split the output of *N* MCMC samples into *L* batches each of length *I*, with *I* chosen large enough that the batch means have low serial correlation, and then estimate *V* using the variance of the batch means. In our simulation examples, the number of MCMC samples, *N*, is 5000 and thus we have used *L* = 50 and *I* = 100. The procedure is given below in steps:

1. Compute the mean of the function of interest (for us, it is the vector of discrepancies at *J* communities) within each batch. That is, for *l* = 1,…*,L*,
  $${\hat{\mu }}_{l}=\frac{1}{I}\sum_{i=(l-1)I+1}^{lI}d_{i}$$
2. The overall mean is then given as $\hat{\mu }=\frac{1}{L}\sum_{l=1}^{L}{\hat{\mu }}_{l}$.
3. Note that $\sqrt{I}\left({\hat{\mu }}_{l}-\hat{\mu }\right),l=1,\ldots ,L$ are approximately independently distributed as *N*_*J*_ (**0***,V* (**D**_*M*_)).
4. Thus, *V* (**D**_*M*_) can be estimated by *V* as

$$\hat{V}=\frac{I}{L-1}\sum_{l=1}^{L}{\left({\hat{\mu }}_{l}-\hat{\mu }\right)}^{2}.$$

## 3. A simulation study

In this section, we study our formulation in the context of HIV epidemic models. We start off this section by describing the model, and then we discuss the simulation settings and the results.

### 3.1. An HIV transmission model

Antiretroviral therapy (ART) has been shown to reduce the infectiousness of HIV infected persons, but only after HIV testing and diagnosis, linkage to care, and successful viral suppression (Hallett et al., 2009; Johnson et al., 2013). Thus a large proportion of HIV transmissions may occur during a period of high infectiousness in the first few months after infection, and is perceived as a threat to the impact of HIV “treatment-as-prevention” strategies. Woods et al. (2018) considered a mathematical model (*M*_*W*_) of HIV epidemics in men through heterosexual contacts, to investigate and explore the population health implications of investing in evidence generation activities such as clinical trials, surveillance programs and health system performance measurement. The model is based on features of a generalized HIV epidemic in sub-Saharan Africa (SSA), and thus can be used as a simplified representation of HIV epidemiology in SSA to evaluate the HIV prevention and treatment strategies available there. More specifically, the model projects how the proportion of early transmission affects the impact of ART on reducing HIV incidence. It includes different stages of HIV infection, sexual mixing, and changes in risk behavior over the epidemic.

**Fig. 3.**
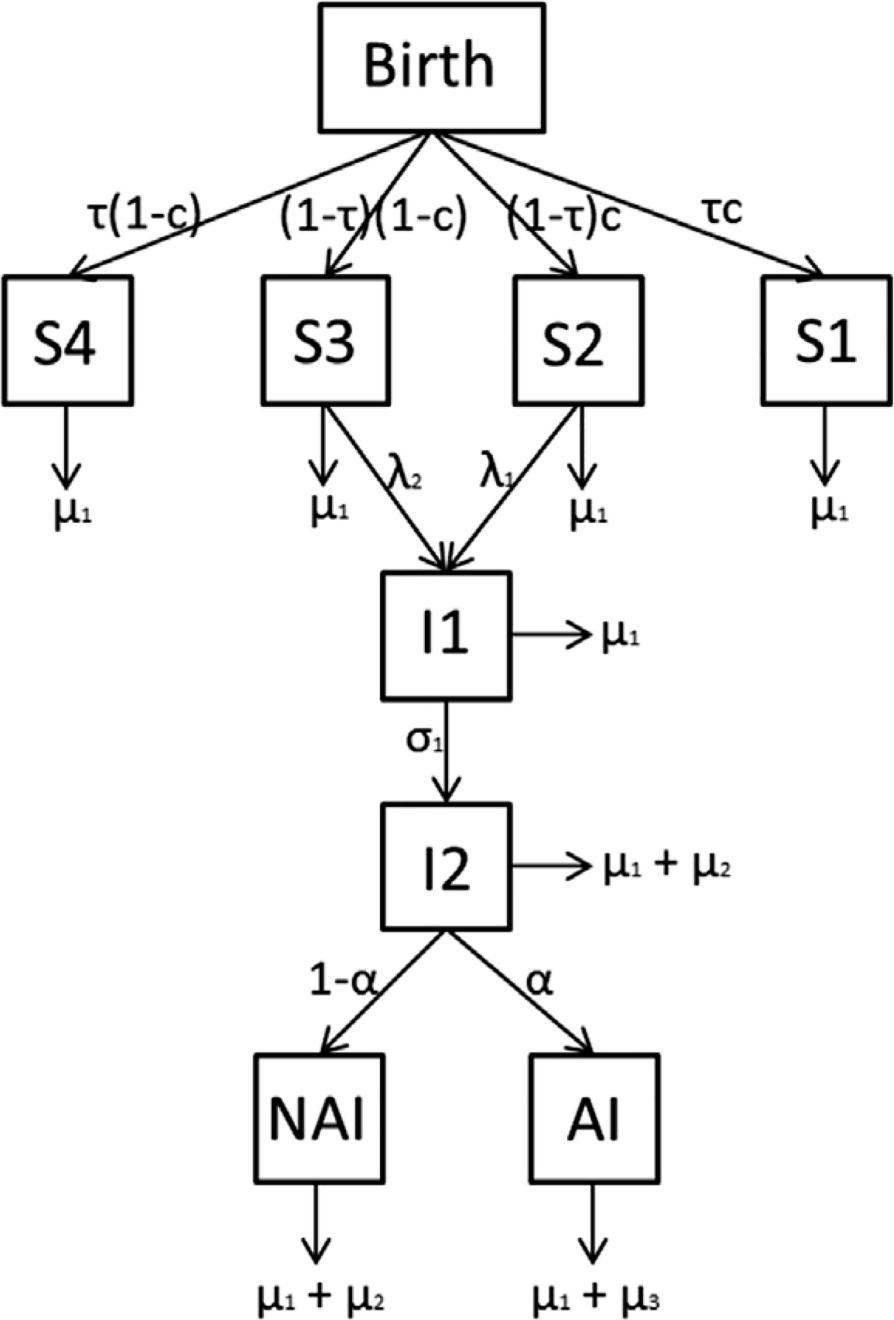
**Model Diagram,** *S*1 denotes circumcised and susceptible men who are not at risk of infection, *S*2 denotes circumcised and susceptible men who are at risk of infection, *S*3 denotes uncircumsized and susceptible men who are at risk of infection, *S*4 denotes uncircumcised and susceptible men who are not at risk of infection, *I*1 denotes men in the first HIV infection state, *I*2 denotes individuals in the second (late stage) infection state, *AI* denotes infected men who will receive the antiretroviral therapy (as part of the intervention package) in 2015, and *NAI* denotes infected men who will never receive ART or other interventions.

The model *M*_*W*_ (see Fig. 3) simulates HIV prevalence in a hypothetical population since 1980, in six different communities (see model diagram in Fig. 3). Prevalence of HIV in each community in 2013 forms the (scalar) community-specific parameter *x*_*k*_ for that community (*k* ∈ {1,…,6}), given as **x**_0_ = {0.11,0.35,0.001,0.03,0.24,0.15}. The model estimates HIV transmission in each community, with parameters fit so that the prevalence in the model in 2013 is as specified in the input prevalence data (the community-specific parameter). If any intervention is applied within the study period, the model can project the effect of this intervention over the next period, compared to the scenario when no intervention is applied. In our hypothetical case, we assume that a combination intervention program is initiated in 2015, and the HIV prevalence is simulated up to 2030. The interventions that are modeled through this combination package in the model are (i) enhanced antiretroviral therapy (ART), which reduces the likelihood that a positive individual will transmit infection and the mortality rate from late-stage infections (ii) behavioral interventions (for example, counseling on condom use or other safe sex practices), which acts to modulate the force of infection; and (iii) medical male circumcision (MMC), which reduces the risk of men to acquire HIV infection. Model equations can be found in the Appendix (and also in Woods et al., 2018). There are 10 global parameters (same for all communities) in the model integrated in the model structure, namely,

1. The reduction in the rate of transmission from individuals on ART, *ε*_*A*_.
2. The reduction in the risk of acquisition of HIV in circumcised men, *ε*_*C*_.
3. The rate of leaving the population (due to aging out or death due to caused unrelated to HIV), *μ*_1_.
4. The population growth rate, *ε*.
5. The rate of progression from infected state *I*1 to *I*2, *σ*_1_.
6. The mortality rate due to HIV in late stage positive individuals in *I*2, *μ*_2_.
7. The mortality rate due to HIV for individuals on ART, *μ*_3_.
8. The rate of transmission of HIV (per partnership), *β*(*t*), that controls the forces of infection, *λ*_1_ and *λ*_2_.
9. Background prevalence rate of HIV in the population in 1980, *p*_*ini*_, considered same for all communities.
10. The proportion of people assumed to be already circumcised in 2013, same for each community, *c*.

The parameter *τ*, denoting the proportion of men entering the population at a given community who are not at risk, is fitted (using least squares) separately for each community, based on the population dynamics of that community at the time when the community-specific parameter was recorded (in our case prevalence data for that community in 2013). Note that *τ* is not considered as community-specific or global parameter, as it is not an input for the model but rather it is fitted based on the input (community-specific and global) parameters. The parameters *ε*_*A*_ and *ε*_*C*_ directly affect the not-at-risk parameter *τ*, as well as the forces of infection, *λ*_1_ and *λ*_2_. The prevalence of HIV, as well as progression and mortality rates due to the disease at a given time *t* is determined by the (time varying) rate of transmission parameter, *β*(*t*), and the proportion of the population who are not at risk, *τ*. As mentioned before, the model was originally calibrated to HIV epidemics data from SSA, and the best point estimates for these parameters were calculated. We denote this estimate vector by *θ*_0_.

### 3.2. The analysis setup

Before we can apply the Bayesian validation procedure on *M*_*W*_, it is important to correctly define the response of interest, one which can give us a substantial idea about the fit of the model. In this analysis, the response was chosen to be the HIV prevalence in each community at the beginning of the year 2020, 5 years after the interventions were initiated in these communities. Next we need to define the distributions $G_{X_{k}}$ and $H_{\Theta }$ for *X*_*k*_*, k* = 1,…,6, and *Θ* respectively. To simplify things, we assume that there is no estimation error in **x**_0_ = {*x*_1_,…*,x*_6_}, that is, the available information on the HIV prevalence in 2013 in these 6 communities reflect the actual truth, or in other words, *X*_*k*_ := *x*_*k*_. We next define priors on the model parameters. Since all of the parameters are proportions between 0 and 1, we consider a logit-Gauss prior in our analysis, that is, logit(*Θ*) ~ *N*(*μ,σ*^2^), with *μ* := logit(*θ*_0_), and *σ*^2^ := *p*∕(*θ*_0_(1 − *θ*_0_)), for a given scale parameter *p*.

For a given instance *Θ* := *θ* and a given timepoint within 1980 and 2030, the model *M*_*W*_ outputs the HIV prevalence vector $\left\{M_{W}\left(x_{1},\theta \right),\ldots ,M_{W}\left(x_{6},\theta \right)\right\}$. We assume that HIV prevalence in 2020 for community *k* can be estimated through a simple random sample of size *n*_*k*_ drawn from the population in question. Thus, given a true HIV prevalence of *y*_*k*_ at community *k* in 2020, we have an unbiased estimate ${\hat{y}}_{k}$, which can be written as ${\hat{y}}_{k}=\frac{Z_{k}}{n_{k}}$, where $Z_{k}\sim B\left(n_{k},y_{k}\right)$ with sampling variability $Var\left({\hat{y}}_{k}\right)=\frac{y_{k}\left(1-y_{k}\right)}{n_{k}}$. We chose a sample size *n*_*k*_ = 2000 for each community in our simulations. Since prevalence is a proportion between 0 and 1, the HIV prevalence values are logit transformed, so that *D*_*M*_(*x,θ*) is distributed over the entire real line. We assume Eq. (5) from Section 2 hold with *M* = logit(*M*_*W*_ ) and *X*_*k*_ := *x*_*k*_, where,

1. $M\left(x_{k},\theta \right)=\mathrm{logit}\left(M_{W}\left(x_{k},\theta \right)\right),$
2. $y_{R,k}=\mathrm{logit}\left(y_{k}\right),$
3. $y_{F,k}=\mathrm{logit}\left({\hat{y}}_{k}\right),$

for *k* = 1,…,6. Note that due to this transformation, $\lambda_{F}\left(y_{R,k}\right)=1/Var\left(y_{F,k}\right)=n_{k}\frac{e^{y_{R,k}}}{{\left(1+e^{y_{R,k}}\right)}^{2}}$, and also that $\hat{\lambda_{F}\left(y_{R,k}\right)}=\lambda_{F}\left(y_{F,k}\right)$.

As discussed in Section 2.2.3, we assume a GaSP prior for the discrepancies **D**_*M*_. For the Bayesian analysis, we can specify priors on the GaSP hyperparameters, *μ*_*d*_, *β*_*d*_ and *λ*_*d*_, as well. However, due to limited data availability, and owing to the fact that no direct data about model discrepancies are available, introducing too many hyperparameters in the model can increase collinearity between the GaSP parameters. Thus, we allow only *λ*_*d*_ to be stochastic, and fix the rest of the hyperparameters at reasonable values, following recommendations of Bayarri et al. (2007), and as discussed in Section 2.2.3. This is achieved in the following manner: the vector $\left\{y_{F,1}-M\left(x_{1},\theta_{0}\right),\ldots ,y_{F,6}-M\left(x_{6},\theta_{0}\right)\right\}$ is treated as a realization from a multivariate normal with constant mean vector *μ*_*d*_ and covariance matrix $C_{d}\left(z_{i},z_{j}\right)/\lambda_{d}+\Lambda_{F}^{-1}$, where *z*_*i*_ = (*x*_*i*_*,θ*_0_) and *Λ*_*F*_ is a diagonal matrix with *k*th diagonal entry *λ*_*F*_ (*y*_*F,k*_). We use standard GaSP fitting software to obtain MLE estimates ${\hat{\beta }}_{d}$, that are used as the fixed value for *β*_*d*_ in this analysis. The mean function *μ*_*d*_ is fixed at 0. We assume an inverse gamma prior for *λ*_*d*_ with shape parameter $\alpha_{\lambda_{d}}=1$ and scale parameter $\beta_{\lambda_{d}}=5{\hat{\lambda }}_{d}$ where ${\hat{\lambda }}_{d}$ is the MLE estimate of *λ*_*d*_ obtained from the GaSP analysis described above, following suggestions from Bayarri et al. (2007). For the Metropolis Hastings algorithm, we also specify proposal distributions for unknown random variables, namely,

- A Gaussian distribution for *D*_*M*_(*x,θ*).
- A Beta distribution for *Θ* (since all are quantities between 0 and 1).
- An Inverse Gamma distribution for *λ*_*d*_.

### 3.3. The simulation scenarios

**Fig. 4.**
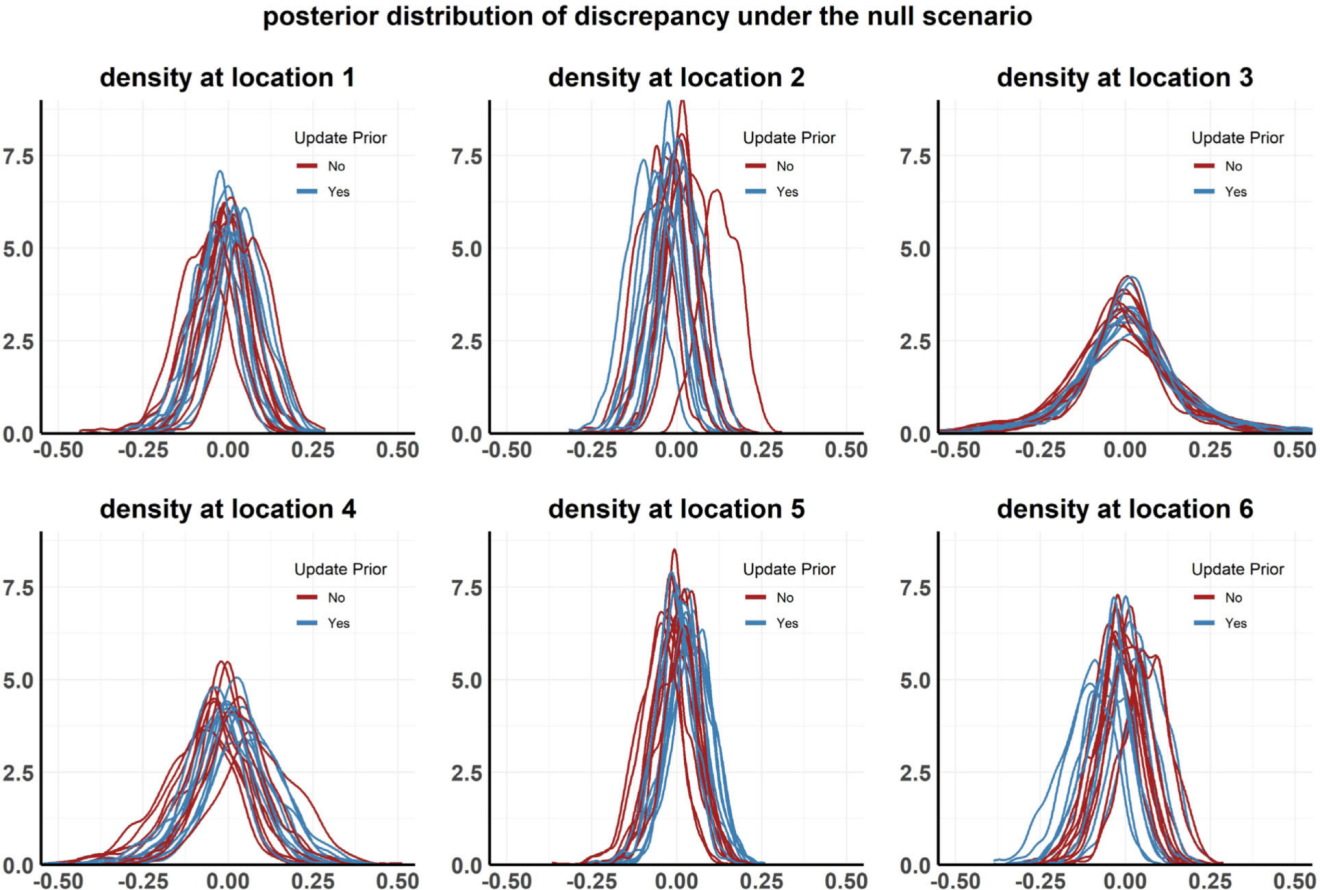
**Setting 1:** Posterior distribution of discrepancy in the null scenario for 10 MCMC chains, each run with a different realization of the observed data.

**Fig. 5.**
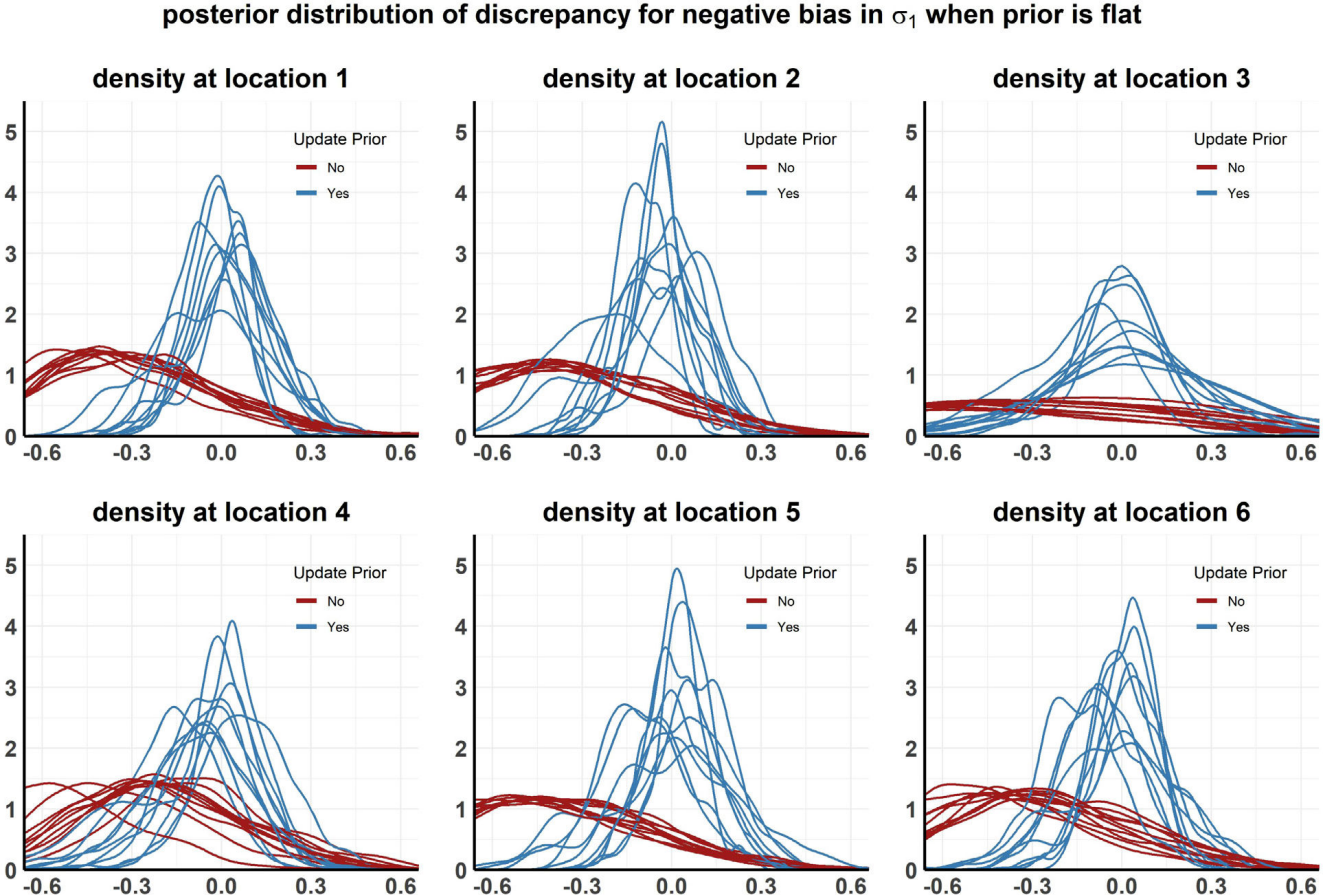
**Setting 2:** Posterior distribution of discrepancy when *σ*_1_ is negatively biased and the prior width is **flat** under 10 MCMC chains, each run with a different realization of the observed data.

**Fig. 6.**
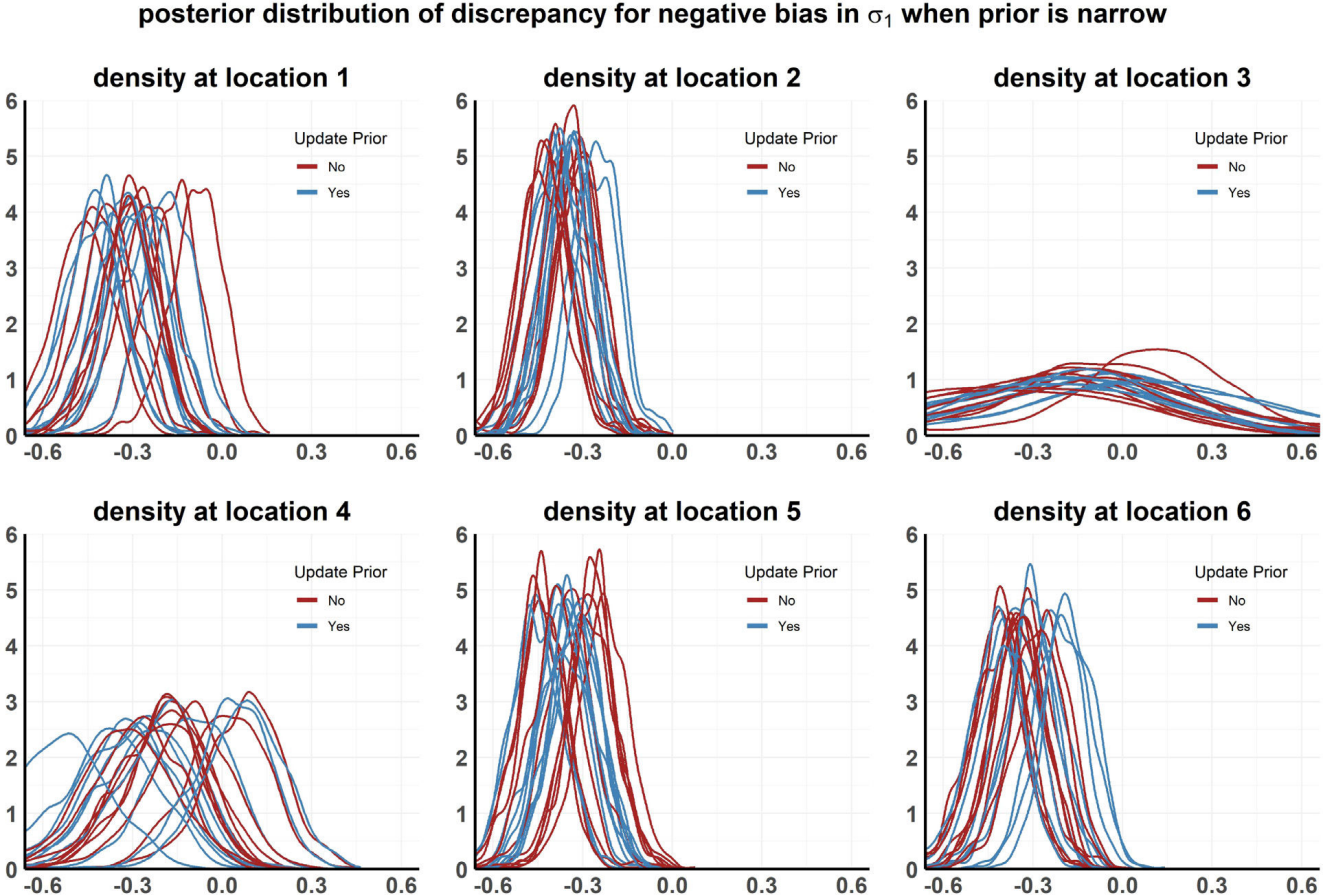
**Setting 2:** Posterior distribution of discrepancy when *σ*_1_ is negatively biased and the prior width is **narrow** under 10 MCMC chains, each run with a different realization of the observed data.

**Fig. 7.**
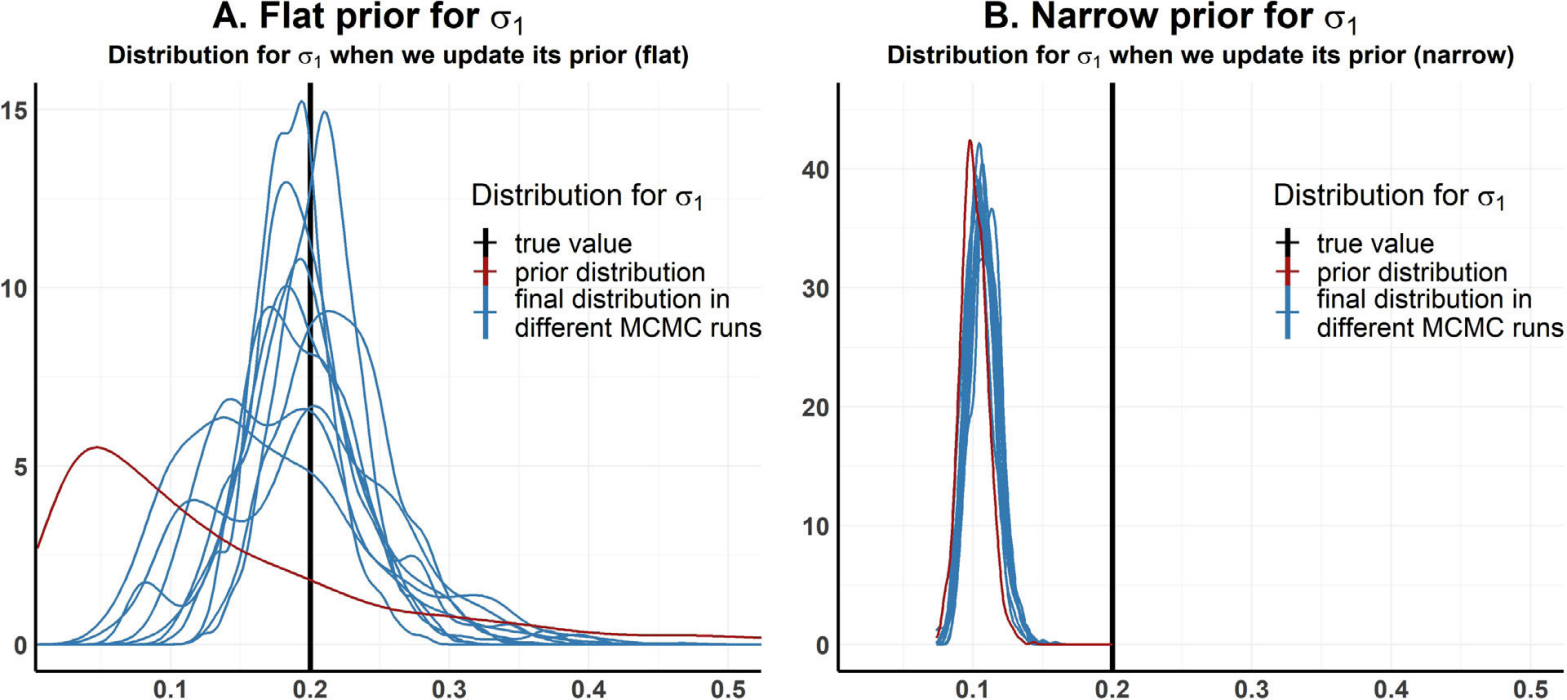
**Setting 2:** Posterior distribution of negatively biased *σ*_1_ when we **update** $H_{\Theta }$ and the prior width is **(A) flat** and **(B) narrow** under 10 MCMC chains, each run with a different realization of the observed data.

We evaluate the model *M*_*W*_ under different scenarios, given by the following:

1. **Setting 1: The Null scenario:** The model is completely accurate, that is, both the model structure *M* and the parameter set *θ*_0_ are accurate, that is, *y*_*R,k*_ = *M*(*x*_*k*_*,θ*_0_), for *k* = 1,…,6. The priors $H_{\Theta }\left(\cdot ;\eta_{\Theta }\right)$ with hyperparameters $\eta_{\Theta }=\left\{\theta_{0},\eta_{\Theta }^{*}\right\}$ reflect a valid quantification of the uncertainty regarding *Θ*. Plot for this setting is given in Fig. 4.
2. **Setting 2: Faulty prior information on *Θ***: The model structure *M* is accurate, but information collected on one or more of the parameters is erroneous. To create this scenario, we start off with the assumption that *θ*_0_ is still the true parameter vector, but we calibrated the model at an incorrect value $\theta_{0}^{*}$, which is created by perturbing some of the elements of *θ*_0_. The priors are now given by $H_{\Theta }\left(\cdot ;\theta_{0}^{*},\eta_{\Theta }^{*}\right)$. In this scenario the following holds for each community *k*.
  $$y_{R,k}=M\left(x_{k},\theta_{0}\right)\;\;\;\;\;\;\;\;\;\;\;=M\left(x_{k},\theta_{0}^{*}\right)+M\left(x_{k},\theta_{0}\right)-M\left(x_{k},\theta_{0}^{*}\right)\;\;\;\;\;\;\;\;\;\;=M\left(x_{k},\theta_{0}^{*}\right)+D^{1}\left(x_{k},\theta_{0}^{*}\right)$$
  In this case, we should be able to falsify the model with running only step 1 of Section 2.3.3. Running step 3 however should recalibrate the parameter set. We can evaluate the accuracy of the parameters after the recalibration step.
  To simplify the analyses, in this scenario, we will consider priors only on the faulty parameters, while keeping the others fixed at their estimates (given in *θ*_0_). This is, similar to what mentioned in Section 2.3.3, an effective simplification of step 3 of running the analysis, where we update the parameter set in multiple iterations, starting off with ones that we are most uncertain about while keeping others fixed at their priors or point estimates, and then moving onto the next batch if the validity of the model is not still achieved. Plots for this setting are given in Figs. 5–7.
3. **Setting 3: Faulty model structure:** The model structure itself is wrong, which means that *M*(*x,θ*) does not correctly specify the reality, even if efforts are made to recalibrate the model in its current form with its given community-specific and global parameters. We create this scenario by producing a reality which is a distortion of the model outputs, that is, we define the alternative (true underlying) model structure as (1+*c*)*M*_*W*_ instead of the presumed structure *M*_*W*_, meaning we add 100*c*% bias to the untransformed model outputs, where *c* is a constant. Thus, we can write $y_{R,k}=M\left(x_{k},\theta_{0}\right)+D^{2}\left(x_{k},\theta_{0}\right)$, where

$$D^{2}\left(x_{k},\theta_{0}\right)=\mathrm{logit}\left((1+c)M_{W}\left(x_{k},\theta_{0}\right)\right)-\mathrm{logit}\left(M_{W}\left(x_{k},\theta_{0}\right)\right).$$

**Fig. 8.**
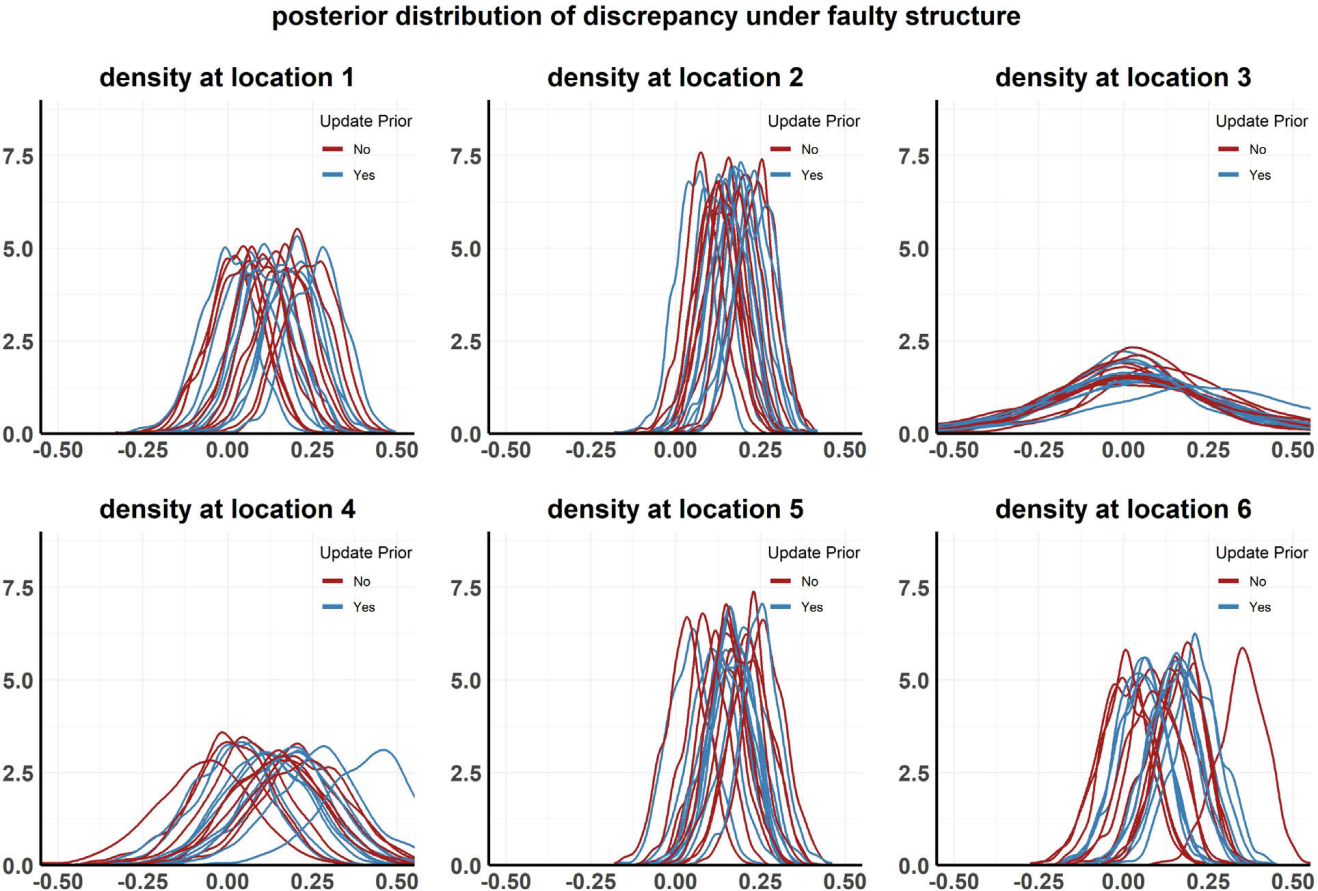
**Setting 3:** Posterior distribution of discrepancy under faulty model structure for 10 MCMC chains, each run with a different realization of the observed data.

In this case, we should be able to falsify the model with running either of the steps 1 and 3 from Section 2.3.3. Plot for this setting is given in Fig. 8. The value of *c* chosen for this exercise is *c* = 0.25.

### 3.4. Results

The results from the above analyses are presented in Figs. 4–8 and in Table 1. Each plot, except Fig. 7, shows the posterior distribution of the discrepancy $D_{M_{W}}\left(x_{k},\Theta \right)$ for communities *k* = 1,…,6 under the different simulation scenarios. Fig. 7 shows the posterior distribution of the parameter *σ*_1_, the rate of progression from infected state *I*1 to *I*2, with and without performing the recalibration step under setting 2 (Faulty prior on *Θ*). The analyses are repeated for multiple realizations of the observed data, resulting in different density curves for the discrepancy in each community for each such realization. In Figs. 4–8, 10 such curves are presented for each simulation scenario. We also calculate the posterior tail probability (as discussed in Section 2.3.4) for each scenario, aggregated over the different MCMC runs, and present the results in Table 1. Some of our observations can be summarized as the following:

#### 3.4.1. Null case: both model and priors are accurate

Under the first simulation setting (the null scenario), the discrepancy for all six communities are centered around 0 both when $H_{\Theta }$ is not updated, and also when we do update it (see Fig. 4). This is expected, since the discrepancy distribution should be centered around 0 when model assumptions are satisfied. However, some individual runs are not centered at 0 at all communities, even after updating — this might be because in those runs, the sampling errors for the communities in question are large.

Updating the prior, increased ${\hat{p}}_{TP}$ from 0.83 to 0.88 Table 1. Under *H*_0_, *p*_*T P*_ should eventually converge 1, given an infinite number of samples. Under finite sample setting, the posterior mean will never be exactly at **0** due to both random fluctuations in the MCMC algorithm and the prior on *λ*_*d*_, and hence the estimated values ${\hat{p}}_{TP}$ will always be less than 1. This provides a benchmark for how good we should expect a model to perform with respect to this metric, given that a ‘perfect’ model scores about 0.85 here.

#### 3.4.2. Recalibrating faulty priors

Under the second simulation scenario, the prior for *σ*_1_, the rate of progression from infected state *I*1 to *I*2, is misspecified downwards by a margin of 50%. We consider two types of faulty priors, (i) Flat — logitgauss prior with scale *p* = 0.1 (Fig. 7A), (ii) Narrow — logitgauss prior with scale *p* = 0.01 (Fig. 7B). Although both of these priors for *σ*_1_ are shifted by the same amount, the flat prior assigns a substantial probability to the true value (black vertical line in 7 than the narrow prior, where it assigns no probability at all).

For both the narrow and flat prior, the model discrepancy is biased substantially away from zero in almost every community when the priors are not updated (as seen in Figs. 5 and 6). However the posterior distribution of *D*_*M*_ includes zero with a higher posterior tail probability in the flat prior than in the narrow prior. This is reflected in the moderately low *p*_*T P*_ of 0.63 for the flat prior, compared to 5.3 × 10^−4^ for the narrow prior.

When the prior of *σ*_1_ is updated in the MCMC analysis, we can obtain a posterior distribution (for *σ*_1_) centered around the true value only it is in the support for the faulty prior originally (Fig. 7). As a result, the posterior distribution of the discrepancy shifts towards 0 in the flat prior but not in the narrow prior (see Figs. 5 and 6), after the recalibration step is performed. After recalibration, the *p*_*T P*_ of the flat prior improves to 0.81, which is comparable to the null case. On the other hand, for the narrow prior, *p*_*T P*_ only improves to 0.088. Thus, even though the model structure is correct, whether we are able to recalibrate the model and verify its validity against the collected data can depend purely on the quantification of uncertainty in the stored value of its parameters, and whether the support of their stored stochastic information contain their true values.

#### 3.4.3. Faulty model structure

Under the third simulation setting, the underlying model structure itself is incorrect, producing biased model outputs, and as a result, the discrepancy is expected to be centered away from 0 even when updating the prior distributions $H_{\Theta }$ for all parameters. Even though that is what we overwhelmingly see in Fig. 8, and that most simulations show a positive bias, one or two curves still appear to be centered around 0. So for those individual simulations, there are not any perceivable difference between the results and model predictions, even though the underlying model structure is faulty. Moreover, this mean shift is often less visible for some communities than others, for example in the case of community 3, which had the lowest prevalence in 2013. Since this scenario was created by inflating the model outputs by 25%, very little discrepancy was introduced in this community, and thus if we were to make a decision about whether the posterior distribution for the discrepancy is centered away from 0 or not, based on individual Bayesian credible intervals at 5% level of error, the hypothesis may be rejected for some communities, but not for others. In addition, for communities with very low prevalence, the sampling error is much higher, and can sometimes dominate the actual discrepancy in the outputs. Under faulty model scenario, ${\hat{p}}_{TP}$ values are low, whether we update the priors or not, but one thing that we notice from Table 1 is that updating the priors (recalibrating) always results in higher values of ${\hat{p}}_{TP}$.

## 4. Discussion

In this article, we outline a framework for evaluating the predictions of complex epidemiological models and describe experiments that can be used to test them. We propose assessing models by calculating the posterior distribution of the model discrepancy using a Bayesian framework. This allows for rapid identification of communities and/or subgroups for which the model performs poorly, and allows for an overall (or locally for each community) goodness of fit evaluation using the posterior tail probability. This methodology can then be systematically applied to update model priors to improve fit.

We apply this framework to a simple model of a heterosexual HIV epidemic, *M*_*W*_, which was created to investigate how the proportion of early transmission affects the impact of ART on reducing HIV incidence. We test the model under various set of assumptions, including the null scenario when all the assumptions are satisfied, along with alternate scenarios when one or more of those assumptions fail to hold, and discuss the scope of recalibration of its parameters when the model is false.

One interesting finding is the existence of ‘uninformative communities’, that is, communities that do not provide information on model validity. In our case, community 3 acts in this way. It had very low prevalence initially, and also at each of the subsequent time points. Looking at the discrepancy plots for community 3 in Figs. 4–8, it becomes obvious that even if there is true discrepancy in the model output, due to a faulty model structure or because one or more of its parameter distributions are wrongly estimated, there is little or no visible sign of that in any of these plots, that is, most of the density plots can be seen to be centered around 0. If anything, the posterior distributions show a higher variability than that at other communities, hinting at the uninformativeness of this particular community.

The complexity of epidemiologic models creates a robustness that makes it quite challenging to falsify them. For example, in the posterior distribution plots for the discrepancy for the faulty model scenario (see Fig. 8), we see that even though most simulations show a positive bias, one or two of the curves do appear to be still centered around 0. So for those individual simulations, there are not any perceivable difference between the results and model predictions, even though the underlying model structure is faulty. Moreover, this mean shift is often less visible for some communities than others (like in the case of community 3), and hence if we were to make a decision about whether the posterior distribution for the discrepancy is centered away from 0 or not, based on individual Bayesian credible intervals at 5% level of error, the hypothesis may be rejected for some communities/subgroups, but not for others. Although these individual inferences are crucial to figure out for which communities the model fails, it is also important to make inference on the overall strength of the model, that is, to aggregate the inferences over these different communities/subgroups. The measure, *p*_*T P*_, defined in Section 2.3.4 based on Mahalonobis distances, helps mitigate that issue somewhat. Delineating faulty model structure from insufficient calibration is a crucial aspect of model validation. This motivated our focus on these scenarios separately.

The methods developed here are for a fixed point of time at which model outputs are recorded and compared with external data. However, these methods can potentially be extended to incorporate comparison of model projections at multiple time points with real-time data as it becomes available over time. For this, one needs to also account for inter-person correlation and time series effects, and it might be worthwhile to pursue this extension in future research. Also note that the (prior) distribution for *λ*_*d*_, the precision parameter for **D**_*M*_ was chosen in a data-driven manner, and the rest of the hyperparameters for the GASP prior on **D**_*M*_ were either fixed at pre-specified or data-driven values. It might be an interesting exercise to conduct a sensitivity analysis to explore the effect of this formulation on the analysis, for example by considering non-data-driven priors for *λ*_*d*_ with varying width, and see how that affects the results.

Note that there can be many different ways a model can have a faulty structure. A faulty structure for an ODE model means that mechanisms and processes which are essential for the population and transmission dynamics are not properly represented in the model. This can be due to multiple factors, including (i) not capturing essential confounders in the model, (ii) not accounting for human mobility patterns in the ODEs, (iii) unexpected events (for example a pandemic, intervention rollout, changes in standards of care) that might render the model structure outdated. Incorporating those factors in the model requires extensive structural changes which were outside the scope of this project. Instead, we assumed that whenever the model structure is wrong, the final effect will necessarily be seen in the predictions, which will be biased estimates (the bias coming from the faulty structure) of the reality. Hence, we decided to mimic the faulty structure by introducing bias in model outputs, without discussing the source of the bias, which can be due to any of the above reasons, or other sources.

Also note that from Section 2, our methods will continue to work for any model of the form *y* = *M*(*x, θ*), where *M*(·, ·) is essentially a function with arguments *x* and *θ*. Thus, the Bayesian validation framework will work for other types of models such as network models and stochastic agent-based models, as long as their parameter sets *x* and *θ* have concrete forms and are estimable (identifiable). However, one limitation the Bayesian validation framework is the fact that the MCMC analysis relies on multiple model runs over different instances of *x* and *θ* generated from the priors. ODE models are generally simple to run and computationally less expensive compared to other models (like agent-based models, network models), which might make the validation procedure extremely computationally burdensome, and therefore, infeasible to run in practice.

It is also worthwhile to mention that in the context of HIV transmission models, reduction in HIV incidence is a better choice as an outcome of interest for evaluation of the impact of real-world interventions. Although the HIV transmission model that we use in our simulation settings was designed to project HIV prevalence, our methods are applicable to modeling analyses where incidence is being estimated. In addition, we note that while incidence is a more desirable metric than prevalence, it is also much harder to estimate than prevalence.

Given a model structure and its parameter priors in their current states and the observed data, ‘optimal’ discrepancy is achieved through re-calibration of its parameters in the Bayesian validation framework but re-calibration is unlikely to reduce the discrepancy to 0. Rather, re-calibration would ensure the smallest possible discrepancy is achieved based on what we knew before (the model) and what we know now (the observed data). To achieve this ‘optimal’ discrepancy, our suggestion is to re-calibrate the parameters in steps, possibly one at a time, where the order can be determined by running sensitivity analyses to gauge the influence of each of these parameters on the final model outputs, and then updating the parameters in order of importance.

For parameters that have identifiability issues, model validation will have to endure some of the same challenges that model calibration face. For example, multimodality in a parameter distribution might indicate presence of latent groups or other structural issues in the model, which may or may not result in multimodality in the discrepancy distribution as well, all of which are important issues to consider in model validation, and we believe that the Bayesian validation framework can be used to diagnose these issues further. As far as the posterior tail probability is concerned, it should be noted that the posterior tail probability alone should not be the only aspect considered in making a decision on model validation, and other visual aspects like multimodality suggesting presence of latent groups or other issues should be taken into consideration as well. Multimodality in the discrepancy distribution may affect the posterior tail probability, for example in the scenario when the discrepancy distribution is bimodal, and the two modes of the discrepancy distribution lie on either side of 0, and the mean lies in between and closer to 0, we might obtain high *p*_*tp*_ indicating good model fit when clearly there are structural issues in the model. This is because *p*_*tp*_ in its current form is effective only when the discrepancy distribution is unimodal, as it defined around the mean of the discrepancy distribution assuming unimodality (see Section 2.3.4). So, when the discrepancy distribution is indeed multimodal, it will be more useful to redefine it in terms of the modes of the discrepancy distribution, and run multiple posterior tail probability analyses, one for each mode.

The HIV Modeling Consortium (www.hivmodelling.org) is a large network of mathematical modelers that aims to strengthen the use of models in decision making in HIV. In the past it has brought together models from different groups to quantify and characterize the extent to which different models predict different impacts of the same interventions (Eaton et al., 2015). The project has revealed a large amount of variation in model outputs, which has led to urgent questions being asked about whether models can be validated. To investigate this further, the Consortium developed a protocol for archiving models and their predictions in 2012, and invited researchers to submit their mathematical models for this exercise. With the methods described in this article, these models can be meaningfully tested and validated, and possibly even recalibrated for future use.

## Figures and Tables

**Table 1:** *p_TP_* measures using Mahalonobis distance for (i) the Null scenario (ii) the Faulty model structure and (iii) Faulty prior information on *Θ* with different prior widths averaged over different MCMC runs.

Update *H*_*Θ*_	Null case	Recalibrating faulty prior		Faulty model structure
		Flat prior	Narrow prior	
False	0.837	0.631	5.33e–04	0.148
True	0.886	0.810	0.088	0.212

## Box I.

(10) $$\alpha_{i}=\alpha \left(\theta_{3}^{\left(c\right)},d_{M}^{\left(c\right)},\lambda_{d}^{\left(c\right)}|{\tilde{\theta }}_{1},\theta_{2}^{\left(i\right)},\theta_{2}^{\left(i-1\right)},\theta_{3}^{\left(i-1\right)},d_{M}^{\left(i-1\right)},\lambda_{d}^{\left(i-1\right)}\right)\;=\min\left\{\frac{q_{\Theta_{3}}\left(\theta_{3}^{\left(c\right)}|\theta_{3}^{\left(i-1\right)}\right)q_{d_{M}}\left(d_{M}^{\left(c\right)}|d_{M}^{\left(i-1\right)}\right)q_{\lambda_{d}}\left(\lambda_{d}^{\left(c\right)}|\lambda_{d}^{\left(i-1\right)}\right)P\left(\theta^{\left(c\right)},d_{M}^{\left(c\right)},\lambda_{d}^{\left(c\right)}|y_{F},x_{0},{\tilde{\theta }}_{1},\theta_{2}^{\left(i\right)}\right)}{q_{\Theta_{3}}\left(\theta_{3}^{\left(i-1\right)}|\theta_{3}^{\left(c\right)}\right)q_{d_{M}}\left(d_{M}^{\left(i-1\right)}|d_{M}^{\left(c\right)}\right)q_{\lambda_{d}}\left(\lambda_{d}^{\left(i-1\right)}|\lambda_{d}^{\left(c\right)}\right)P\left(\theta^{\left(i-1\right)},d_{M}^{\left(i-1\right)},\lambda_{d}^{\left(i-1\right)}|y_{F},x_{0},{\tilde{\theta }}_{1},\theta_{2}^{\left(i-1\right)}\right)},1\right\}.$$

## Footnotes

1 We will use *X*_*k*_, *Θ* etc to denote Random Variables, while *x*_*k*_ and *θ* will be reserved to denote observed realizations of such, and/or to denote fixed quantities.

## Appendix A. Details of the modified Bayesian procedure

Here we present the details of the Bayesian analysis under certain modified conditions that we have adopted in our simulation setting. Here, we assume that a) we have high level estimates for the community-specific parameters in each community *k*, that is, *X*_*k*_;= *x*_0*,k*_, and b) *λ*_*F*_ is explicitly known through the design. Denote **x**_0_ = {*x*_0,1_,…*,x*_0*,K*_}.

The multivariate normal density for the collection of all field data *y*_*F,*1_, …, *y*_*F.K*_ can now be denoted by $f(y_{F,1},\ldots ,y_{F,K}|\Theta ,x_{0},\lambda_{F}\left(x_{0}\right)D_{M},\lambda_{d},\mu_{d},\beta_{d},\alpha_{d})$, and the posterior density of the unknowns given the data *y*_*F*_ can be simplified (after removing the fixed quantities from the conditional distributions) as

(9) $$P\left(\Theta ,D_{M}\left(x_{0,1},\Theta \right),\ldots ,D_{M}\left(x_{0,K},\Theta \right),\lambda_{d}|y_{F,1},\ldots ,y_{F,K},x_{0}\right)\;\propto f\left(y_{F,1},\ldots ,y_{F,K}|\Theta ,x_{0},D_{M}\left(x_{0,1},\Theta \right),\ldots ,D_{M}\left(x_{0,K},\lambda_{d}\right)\right)\;\;\times P\left(\Theta ,D_{M}\left(x_{0,1},\Theta \right),\ldots ,D_{M}\left(x_{0,K},\Theta \right),\lambda_{d}|x_{0}\right).$$

## Appendix B. Metropolis Hastings algorithm

The Metropolis Hastings (MH) is an efficient method for sampling of the random variables (*Θ,***D**_*M*_*,λ*_*d*_) from their posterior distribution. We divide our parameters into three classes, *Θ* = {*Θ*_1_*,Θ*_2_*,Θ*_3_}, where (i) *Θ*_1_ are parameters that we want to fix at degenerate values (prior means)${\tilde{\theta }}_{1}$, that is, $\Theta_{1}:={\tilde{\theta }}_{1}$, (ii) *Θ*_2_ are parameters with prior distribution $H_{2}\left({\tilde{\theta }}_{2}\right)$ which we do not want to update, and (iii) *Θ*_3_ are parameters with prior distribution $H_{3}\left({\tilde{\theta }}_{3}\right)$ which we want to update. The posterior distribution that we want to sample from is given as $P\left(\Theta_{3},D_{M},\lambda_{d}|y_{F},x_{0},{\tilde{\theta }}_{1},\Theta_{2}\right)$.

At each step in the MH algorithm, we define a proposal distribution for each of the random variables whose joint posterior distribution is of interest to us. Thus, we assume we have proposal distributions $q_{\Theta_{3}}$, $q_{D_{M}}$, and $q_{\lambda_{d}}$ for *Θ*_3_, **D**_*M*_ and *λ*_*d*_ such that we can draw values $\theta_{3i}\sim q_{\Theta }\left(\cdot |\eta_{0}\right)$, $d_{Mi}\sim q_{d_{M}}\left(\cdot |d_{M0}\right)$, and $\lambda_{di}\sim q_{\lambda_{d}}\left(\cdot |\lambda_{d0}\right)$ given some parameters *η*_0_, **d**_*M*0_ and *λ*_*d*0_. In the first step, we draw initial values $\left(\theta_{2}^{\left(0\right)},\theta_{3}^{\left(0\right)},d_{M}^{\left(0\right)},\lambda_{d}^{\left(0\right)}\right)$ for (*Θ*_2_*,Θ*_3_,**D**_*M*_*,λ*_*d*_) directly from the prior, that is,$\left(\theta_{2}^{\left(0\right)},\theta_{3}^{\left(0\right)},d_{M}^{\left(0\right)},\lambda_{d}^{\left(0\right)}\right)\sim H_{2}\left({\tilde{\theta }}_{2}\right)H_{3}\left({\tilde{\theta }}_{3}\right)P\left(D_{M},\lambda_{d}|x_{0}\right):=P\left(\Theta_{2},\Theta_{3},D_{M},\lambda_{d}|x_{0}\right)$. Then the *i*th iteration of the algorithm is conducted in the following steps:-

1. Generate candidate proposals $\left(\theta_{3}^{\left(c\right)},d_{M}^{\left(c\right)},\lambda_{d}^{\left(c\right)}\right)$ as $\theta_{3}^{\left(c\right)}\sim q_{\Theta_{3}}\left(\cdot |\theta^{\left(i-1\right)}\right)$, $d_{M}^{\left(c\right)}\sim q_{D_{M}}\left(\cdot |d_{M}^{\left(i-1\right)}\right)$, and $\lambda_{d}^{\left(c\right)}\sim q_{\lambda_{d}}\left(\cdot |\lambda_{d}^{\left(i-1\right)}\right)$.
2. Generate $\theta_{2}^{\left(i\right)}$ as $\theta_{2}^{\left(i\right)}\sim H_{2}\left({\tilde{\theta }}_{2}\right)$.
3. Calculate the acceptance probability at the *i*th step, *α*_*i*_, as Eq. (10) given in Box I.
4. Accept the candidate sample $\left(\theta_{3}^{\left(i\right)}:=\theta_{3}^{\left(c\right)},d_{M}^{\left(i\right)}:=d_{M}^{\left(c\right)},\lambda_{d}^{\left(i\right)}:=\lambda_{d}^{\left(c\right)}\right)$ with probability *α*_*i*_ or reject it $(\theta_{3}^{\left(i\right)}:=\theta_{3}^{\left(i-1\right)},d_{M}^{\left(i\right)}:=d_{M}^{\left(i-1\right)},\lambda_{d}^{\left(i\right)}:=\lambda_{d}^{\left(i-1\right)})$ with probability 1 − *α*_*i*_.

After a mandatory burn-in period, the resulting samples (a set of *N* draws) are drawn from the posterior distribution of *Θ*_3_, *λ*_*d*_, **D**_*M*_ (and resultantly (**x**_0_*,Θ*)).

## Appendix C. Model equations for the HIV transmission model *M*_*W*_

The Model diagram in Fig. 3 is described by a set of eight ordinary differential equations described below.

$$\frac{dS_{1}}{dt}=B\tau c-\mu_{1}S_{1}$$

$$\frac{dS_{2}}{dt}=B(1-\tau )c-\left(\lambda_{1}+\mu_{1}\right)S2$$

$$\frac{dS_{3}}{dt}=B(1-\tau )(1-c)-\left(\lambda_{2}+\mu_{1}\right)S_{3}$$

$$\frac{dS_{4}}{dt}=B\tau (1-c)-\mu_{1}S_{4}$$

$$\frac{dI_{1}}{dt}=\lambda_{1}S_{2}+\lambda_{2}S_{3}-\left(\mu_{1}+\sigma_{1}\right)I_{1}$$

$$\frac{dI_{2}}{dt}=\sigma_{1}I_{1}-\left(1+\mu_{1}+\mu_{2}\right)I_{2}$$

$$\frac{dAI}{dt}=\alpha I_{2}-\left(\mu_{1}+\mu_{3}\right)AI$$

$$\frac{dNAI}{dt}=(1-\alpha )I_{2}-\left(\mu_{1}+\mu_{2}\right)NAI$$

where *S*1 denotes circumcised and susceptible men who are not at risk of infection, *S*2 denotes circumcised and susceptible men who are at risk of infection, *S*3 denotes uncircumsized and susceptible men who are at risk of infection, *S*4 denotes uncircumcised and susceptible men who are not at risk of infection, *I*1 denotes men in the first HIV infection state, *I*2 denotes individuals in the second (late stage) infection state, *AI* denotes infected men who will receive the antiretroviral therapy (as part of the intervention package) in 2015, and *NAI* denotes infected men who will never receive ART or other interventions.

The number of individuals entering the population is denoted by *B*, while the rate of individuals leaving the population is denoted by *μ*_1_. The proportion of individuals entering the population who are not at risk is denoted by *τ*, and *c* is the proportion of those entering the population who are circumcised men. *λ*_1_ and *λ*_2_ describe the force of infection in circumcised and uncircumcised individuals respectively. *σ*_1_ describes the rate of progression from infected state *I*_1_ to *I*_2_, and *α* is the proportion of individuals in state *I*_2_ who will receive ART. *μ*_2_ is the rate of death in late stage positive individuals (*I*_2_) and *μ*_3_ is the rate of death for individuals on ART treatment.

The force of infection is described by the following equations:

$$\lambda_{1}=\left(1-\varepsilon_{C}\right)\beta (t)\eta \frac{I_{1}+I_{2}+NAI+\left(1-\varepsilon_{A}\right)AI}{S_{1}+S_{2}+S_{3}+S_{4}+I_{1}+I_{2}+AI+NAI}$$

$$\lambda_{2}=\beta (t)\eta \frac{I_{1}+I_{2}+NAI+\left(1-\varepsilon_{A}\right)AI}{S_{1}+S_{2}+S_{3}+S_{4}+I_{1}+I_{2}+AI+NAI}$$

where *ε*_*C*_ is the reduction in the risk of acquisition of HIV in circumcised men, *β*(*t*) is the rate of transmission, *η* is used to modulate the force of infection to simulate a behavior change intervention, and *ε*_*A*_ is the reduction in the rate of transmission from individuals on treatment. The number of individuals entering the population is described by:

$$B=\left(-\mu_{1}+\varepsilon \right)P$$

where *ε* represents the population growth rate and *P* represents the population size 15 years prior to the current time period. *P* was defined in this way to avoid differences in prevalence caused by interventions affecting the birth rate. The population at the year of the intervention is scaled to ensure that all regions have the same population size at the start of the intervention period.

There is also an additional parameter ‘beta trend parameter’ which is applied in all communities and is used to scale the rate of transmission *β*. This is separate from the other parameters so that it may be incorporated into uncertainty analyses. If time *t* is less than the time when the trend is turned on (*t*_*trend*_), *β* is equal to the initial value specified, if time is greater than *t*_*trend*_, *β* is modified using the beta trend parameter (*ω*).

$$\;\text{If}\;t\leq t_{\text{trend}},$$

β(t)=β,

$$\text{If}\;t>t_{\text{trend}},$$

$$\beta (t)=\beta^{\left(1-\left(\omega \left(t-\left(t_{trend\;}-1\right)\right)\right)\right)}$$

where *t*_*trend*_ corresponds to the year 2015 and as such this modulation only occurs once the fitting has been completed (we fit the model to 2013 prevalence). Both *β* and *ω* are the same across all communities.
